# Spectroscopic Signature of Apoptosis Induced by Venetoclax in Philadelphia-Positive Acute Lymphoblastic Leukemia

**DOI:** 10.1021/acs.analchem.5c07689

**Published:** 2026-08-14

**Authors:** Marta Szewczyk, Patrycja Dawiec, Anna M. Nowakowska, Justyna Jakubowska, Agata Pastorczak, Małgorzata Barańska, Wojciech Młynarski, Katarzyna Majzner, Kinga Ostrowska

**Affiliations:** † Department of Pediatrics, Oncology and Hematology, 37808 Medical University of Lodz, Pomorska 251, 92-513 Lodz, Poland; ‡ Faculty of Chemistry, Department of Chemical Physics, 37799 Jagiellonian University, Gronostajowa 2, 30-387 Krakow, Poland; § Doctoral School of Exact and Natural Sciences, Jagiellonian University, Lojasiewicza 11, 30-348 Krakow, Poland; ∥ Department of Genetic Predisposition to Cancer, 37808 Medical University of Lodz, Czechoslowacka 4, 92-216 Lodz, Poland

## Abstract

Understanding why some leukemia cells undergo apoptosis upon venetoclax (VEN) treatment while others remain refractory is essential for improving therapeutic outcomes. Here, we integrate Raman microscopy, FT-IR imaging, and chemometrics to explore treatment-associated spectroscopic patterns linked to differential VEN response in *Philadelphia*-positive B-cell acute lymphoblastic leukemia (Ph+ B-ALL). Using two BCR-ABL1-positive cell lines with intrinsic differences in VEN sensitivity (BV-173, sensitive; SD-1, resistant), we combined classical biological readouts of apoptosis with multimodal vibrational profiling to characterize global biochemical changes induced by VEN. Sensitive BV-173 cells displayed spectroscopic changes consistent with apoptotic progression, reflected in coordinated alterations in nucleic acids, proteins, and lipids. In contrast, resistant SD-1 cells exhibited a distinct treatment-associated biochemical response profile consistent with nonapoptotic adaptation. Spectroscopic fingerprints enabled differentiation between apoptotic and nonapoptotic response patterns in the cell-line models studied. Our findings demonstrate that vibrational spectroscopy, integrated with chemometrics, may provide a complementary analytical framework for exploratory characterization of treatment-associated biochemical phenotypes in leukemia.

## Graphical abstract

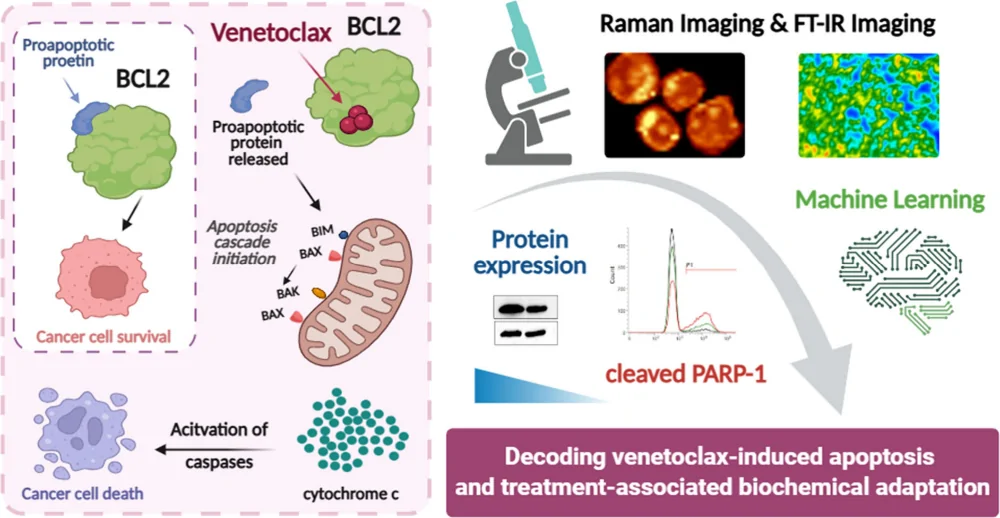

## Introduction

Acute lymphoblastic leukemia (ALL) is a heterogeneous malignancy of immature lymphoid progenitors.^1^ B-cell precursor ALL (BCP-ALL) accounts for approximately 80%–85% of cases.^2^ Among its molecular subtypes, *BCR-ABL1*-positive (Philadelphia chromosome-positive, Ph+) B-ALL is associated with aggressive disease biology driven by constitutive kinase activity of the BCR-ABL1 fusion protein.^3^ Despite the introduction of targeted therapies, relapse remains a major clinical challenge.^4^ Dysregulation of apoptosis plays a central role in therapy resistance, and inhibition of antiapoptotic proteins has therefore emerged as a rational therapeutic strategy. This intracellular process, responsible for maintaining homeostasis and regulating the cell population, is triggered by two independent pathways: exogenous (extrinsic), regulated by death receptor activation, and endogenous (intrinsic) mediated by mitochondria.^5,6^

The mitochondrial pathway of apoptosis is supervised and regulated by members of the B-cell lymphoma (BCL-2) family of proteins, which include pro-apoptotic proteins (BAK and BAX) and antiapoptotic proteins (MCL-1, BCL-2, and BCL-XL).^7^ The BCL-2 protein is deregulated in numerous hematological diseases. Furthermore, its overexpression is associated with resistance to treatment and disease progression. As inhibition of apoptosis is essential for the uncontrolled proliferation of cancer cells, targeting this pathway with venetoclax, a BH3-mimetic inhibitor of B-cell lymphoma 2 (BCL-2), has emerged as a prominent therapeutic strategy.^8^ Venetoclax (VEN), due to its ability to restore apoptotic signaling in cells, has been successfully introduced for the treatment of adult patients with acute myeloid leukemia (AML) and chronic lymphocytic leukemia (CLL).^9^ Currently, this promising BCL-2 inhibitor is being evaluated in other hematological malignancies, including ALL, pediatric AML, and multiple myeloma (MM).^10^ Furthermore, strong synergistic interactions between VEN and other anticancer agents (e.g., the casein kinase 2 inhibitor silmitasertib) have been observed in resistant BCP-ALL cell lines.^9,11^ However, studies on Ph+ and Ph-like ALL revealed significantly reduced sensitivity to BCL-2 inhibition compared with Ph- cells. A potential explanation might be a higher BCL-W and lower BCL-2 expression.^12^ Despite many advantages, resistance to VEN persists, often not fully explained by cancer biology, remaining a major therapeutic challenge. Resistance mainly involves a substitution mechanism in which malignant cells upregulate non-BCL-2 antiapoptotic proteins to replace BCL-2 and bind to BIM. Another pattern involves metabolic changes, especially in OXPHOS and fatty acid metabolism.^13^ Moreover, changes within the mitochondrial structure are considered as potential perpetrators of drug resistance.^14^ The proposed mechanisms for VEN resistance are continually expanding, including new aspects such as epigenetic changes, tumor suppressor gene expression, and the cell cycle, which are being investigated.

Vibrational spectroscopy, especially Raman spectroscopy (RS) and Fourier transform infrared (FT-IR) spectroscopy, is a powerful, label-free tool for analyzing biochemical changes in cancer cells. Recently, these methods, combined with multivariate analysis, have been used to identify leukemia subtypes, characterize genetic abnormalities, and study resistance to chemotherapy and tyrosine kinase inhibitors.^15–19^

In our previous work, we primarily used RS for molecular subtype classification in ALL, including discrimination between B-ALL and T-ALL,^20,21^ assessment of chromosomal alterations and metabolic phenotypes in hyperdiploid B-ALL,^22^ identification of spectroscopic markers associated with KMT2A rearrangements and BCR-ABL1 fusion,^23^ and monitoring of leukemia cell responses to targeted therapy with ruxolitinib.^24^ However, these studies were mainly focused on subtype discrimination or single-modality analysis rather than on functional characterization of stage-specific drug-response phenotypes within the same molecular background.

The integration of complementary vibrational spectroscopy techniques with single-cell analysis enables deeper insight into functional biochemical changes associated with the therapeutic response. In the present study, we shift the focus from leukemia subtype classification toward functional drug response phenotyping within the same molecular background. Specifically, we analyzed stage-specific cellular responses to venetoclax (VEN) in Ph+ B-ALL cells.

By integrating RS and FT-IR imaging within a unified chemometric framework, we characterize coordinated alterations in nucleic acids, proteins, and lipids associated with VEN exposure. Rather than focusing on diagnostic discrimination, our goal was to distinguish spectroscopic patterns associated with apoptotic and nonapoptotic cellular responses within the investigated cell-line models and to identify features accompanying VEN-induced apoptosis.

## Materials and Methods

### Cell Culture and Treatment

Human Philadelphia chromosome–positive (Ph+) B-cell precursor acute lymphoblastic leukemia (BCP-ALL) cell lines BV-173 and SD-1 (DSMZ, Germany) were cultured in RPMI 1640 (Gibco, 21875034) medium supplemented with 10%–20% fetal bovine serum and 100 U/mL penicillin–streptomycin at 37 °C in a humidified 5% CO_2_ atmosphere. Cells were treated with venetoclax (VEN; ABT-199, Selleckchem) at 10 nM and 100 nM for 4 and 24 h. Untreated cells served as controls. Three independent biological replicates were performed for each condition. The cytostatic/cytotoxic effect of VEN was assessed using the MTT assay. The apoptotic response was validated by flow cytometry using PI staining and cleaved PARP-1 detection, as described in Sections 1.1–1.3 of the SI.

### Immunoblotting

Total protein was extracted from BV-173 and SD-1 cells after VEN treatment using RIPA buffer supplemented with protease inhibitors (Thermo Fisher Scientific). Protein concentration was determined, and 30 μg of total protein per sample was separated under reducing conditions on 12% SDS-PAGE gels and transferred to PVDF membranes (Bio-Rad). Membranes were blocked with 5% BSA in TBST and incubated with primary antibodies against selected pro- and antiapoptotic Bcl-2 family proteins (Cell Signaling Technology), followed by HRP-conjugated secondary antibodies. Protein bands were visualized using chemiluminescence (Bio-Rad) and documented with a ChemiDoc imaging system.

### Raman and FT-IR Imaging

Single-cell Raman images were acquired using a WITec Alpha 300 confocal Raman microscope (WITec GmbH, Germany) equipped with a 532-nm excitation laser and a CCD detector. Cells suspended in buffer were placed on CaF_2_ windows and measured using a water-dipping objective (NA = 1.0). Spectra were collected with a spatial resolution of 1 μm, an integration time of 0.5 s per pixel, and a laser power of 50 mW (measured before the objective using the integrated power meter). No additional accumulations were applied. Measurements were performed on fixed cells immersed in buffer to ensure efficient heat dissipation. No photothermal damage or spectral instability was observed. At least 50 cells per condition were analyzed across three biological replicates.

FT-IR measurements were performed using a Varian 620-IR microscope coupled to a 670-IR spectrometer equipped with a 128 × 128 focal plane array detector in transmission mode. Spectra were recorded in the 900–3600 cm^–1^ range at 4 cm^–1^ resolution. For each sample, three infrared images (700 × 700 μm) were collected. Three independent biological replicates were analyzed. A detailed description of cell preparation and the Raman/FT-IR measurement protocols can be found in Sections 1.4–1.6 of the Materials and Methods section in the SI.

### Spectral Preprocessing and Chemometric Analysis

Raman spectra were corrected for cosmic rays (filter size 3, dynamic factor 8) and baseline (third-order polynomial, Project FIVE, WITec). Single-cell average spectra were obtained from hyperspectral maps by using *k*-means cluster analysis (KMCA).

FT-IR spectra were processed in CytoSpec (ver. 2.00.07). Spectra from cell-free regions were excluded using a thickness criterion (1620–1680 cm^–1^). Noise reduction was performed by PCA (15 PCs), followed by Savitzky–Golay smoothing (13 points). Average spectra from defined cellular regions were extracted using a custom Python algorithm (300 spectra per sample; 100 per biological replicate).

Chemometric analysis was performed using PLS-Toolbox Solo (Eigenvector Research). Raman spectra were preprocessed with Savitzky–Golay smoothing (third order, 13 points), EMSC, and mean centering. FT-IR spectra were transformed using a second derivative (Savitzky–Golay, third order, 17 points), followed by MSC and mean centering. Preprocessing parameters were predefined and applied uniformly. Initial PCA analysis performed after conventional preprocessing revealed dominant replicate-associated variance, primarily reflected in baseline-related spectral contributions. Therefore, an additional EMSC-based preprocessing strategy was applied to reduce replicate-associated spectral variability before chemometric analysis. EMSC preprocessing was performed jointly on the complete dataset, using the mean spectrum as the reference, following approaches previously proposed for harmonizing vibrational spectroscopy datasets acquired under varying experimental conditions.

PLS-DA was conducted in the ranges 600–1800 cm^–1^ (Raman) and 926–1760 cm^–1^ (FT-IR). Cross-Validation (CV) used the Venetian blinds method (10 splits; samples per blind = 10). Outliers were evaluated using Q-residuals and Hotelling’s T^2^. Model performance was assessed by sensitivity, specificity, and RMSE.

Permutation testing (200 permutations) verified that class separation exceeded chance-level expectations (Figures S3–S5). The stability of discriminative spectral regions was further evaluated using alternative preprocessing strategies and replicate combinations, with results visualized as VIP heatmaps (Figures S6–S10). Regression vectors and VIP scores were used to identify spectral regions contributing to group differentiation. Since the analysis was exploratory, no parameter tuning based on validation accuracy was performed.

Group-average spectra and standard deviations were calculated at the single-cell level (Table S1). Data visualization was performed with OriginPro 2024b.

## Results

### Response to VEN

Following the comprehensive screening of VEN sensitivity in B-ALL cell lines that was previously described by Fidyt et al.,^25^ we evaluated two cell lines with differential VEN sensitivity: BV-173 (sensitive) and SD-1 (resistant) (Figure 1). Both cell lines represent B-ALL carrying the oncogenic *BCR-ABL1* fusion gene. Treatment with 1 μM VEN did not reduce the viability of the SD-1 cell line. The IC_50_ values calculated from the MTT assay were above 4 μM for the resistant SD-1 cells and 140 nM for the sensitive BV-173 cells, respectively.

**Figure 1:**
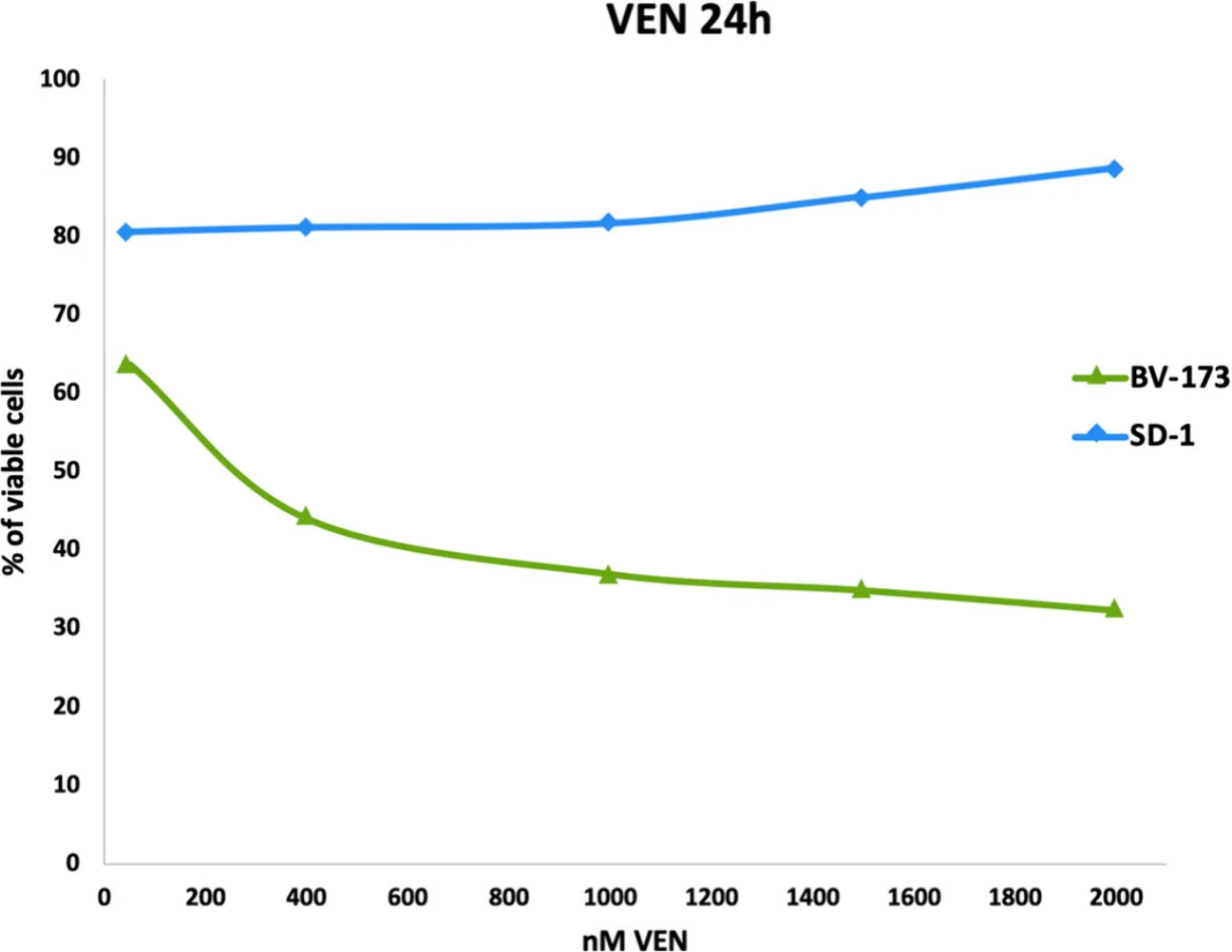
The comparison of leukemic cell lines’ viability after exposure to VEN for 24 h. The viable cells are presented as a percentage of the untreated (control) cells. The plot represents means ± SD.

The results obtained served to design an experiment reflecting different responses to VEN of B-ALL cells to VEN. We applied two exposure time points (4 and 24 h) to VEN across a range of drug concentrations. In the 4-h exposure, we used 10 nM and 100 nM VEN. In the 24-h experiment, exposure to 10 nM VEN was expected to cause approximately a 20% reduction in cell growth. Preliminary biological characterization of cell viability and apoptosis was performed to establish reference conditions for subsequent Raman and FT-IR analyses and validate the expected differential response to VEN.

To achieve optimal conditions for the VEN’s influence on cells, the key elements were the concentration and exposure time. We did not observe cell death at above 20%. The results of PI staining (Figure 2) confirmed SD-1 cells’ drug resistance and BV-173 cells’ sensitivity to VEN, respectively. In the case of BV-173, 4-h treatment with 100 nM VEN did not influence cell viability. However, prolonged exposure to VEN significantly reduced the cell viability. This observation might support the conclusion that 4 h of exposure to VEN is consistent with an early apoptotic response.

**Figure 2:**
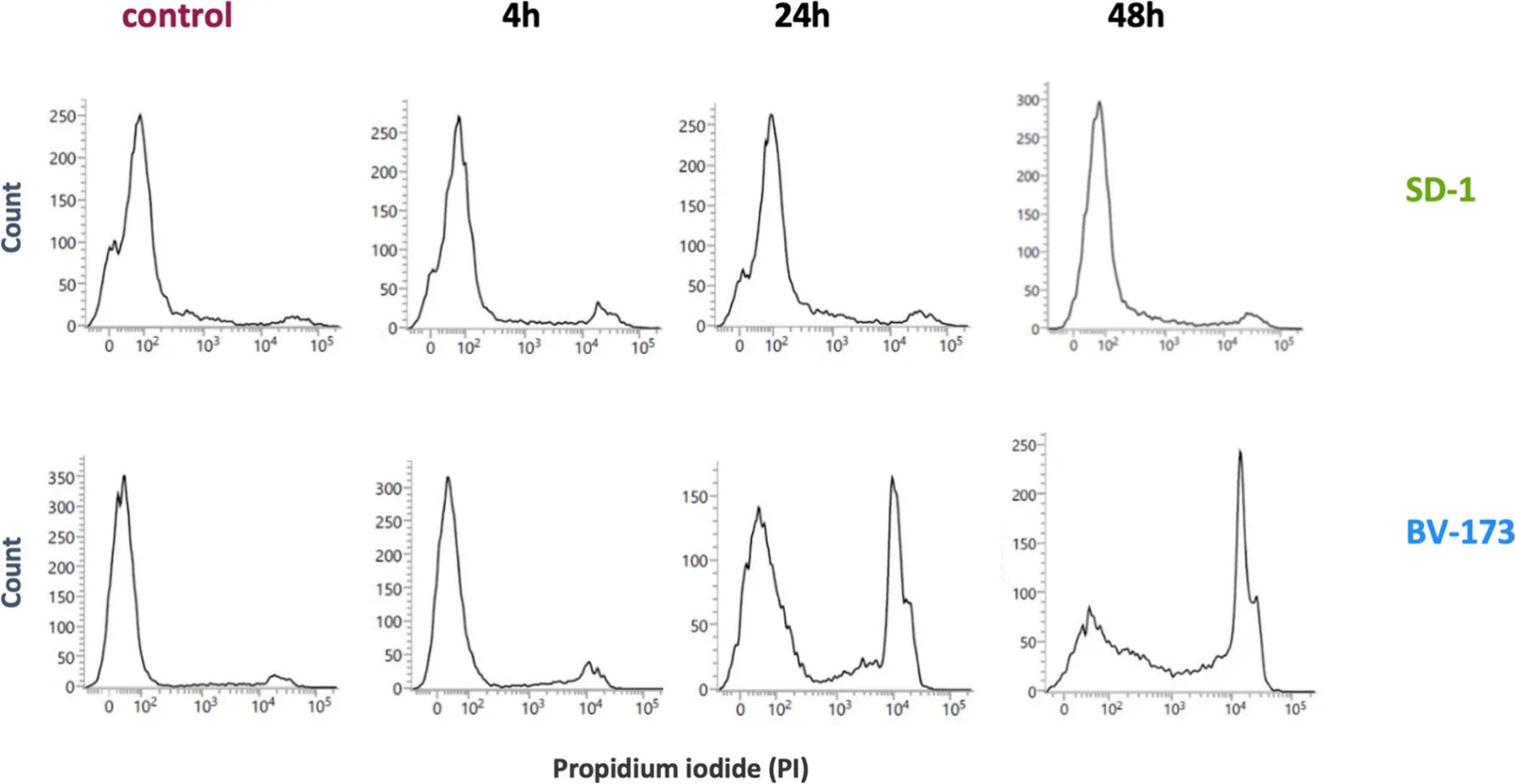
Histograms displaying the effects of exposure to VEN depending on the time of incubation with the drug. Cell death/PI positivity was assessed by flow cytometry. The BV-173 and SD-1 were exposed to 100 nM VEN for 4, 24, and 48 h.

### Cleaved PARP-1 as a Marker of Apoptosis

To validate the apoptotic effect induced by VEN in the cell-sensitive model, we measured cleaved PARP-1 levels by flow cytometry (Figure 3). PARP-1 is a nuclear enzyme involved in DNA repair. Because PARP-1 is also a substrate of caspases, cleavage of PARP-1 by caspases is considered to be a hallmark of apoptosis.^26^ We observed that in BV-173 sensitive cells, signal from cleaved PARP-1 appeared after short-time incubation with VEN (4 h), and the effect was intensified after 24 h of incubation. We did not observe PARP-1 cleavage in SD-1 cells after VEN treatment at any measured time point. Detection of cleaved PARP-1 served as an independent biological validation of VEN-induced apoptosis and defined the reference apoptotic state for a subsequent spectroscopic interpretation. Sensitive BV-173 cells initiate apoptosis even after 4 h of incubation with VEN, illustrating the initial phase of this process, in contrast to the late-stage apoptosis observed after 24 h. The results of PARP-1 cleavage were in accordance with PI staining, which also showed a prominent increase in cell death after 24 h of VEN treatment. The identification of PARP-1 cleavage also served as validation for the Raman measurement.

**Figure 3:**
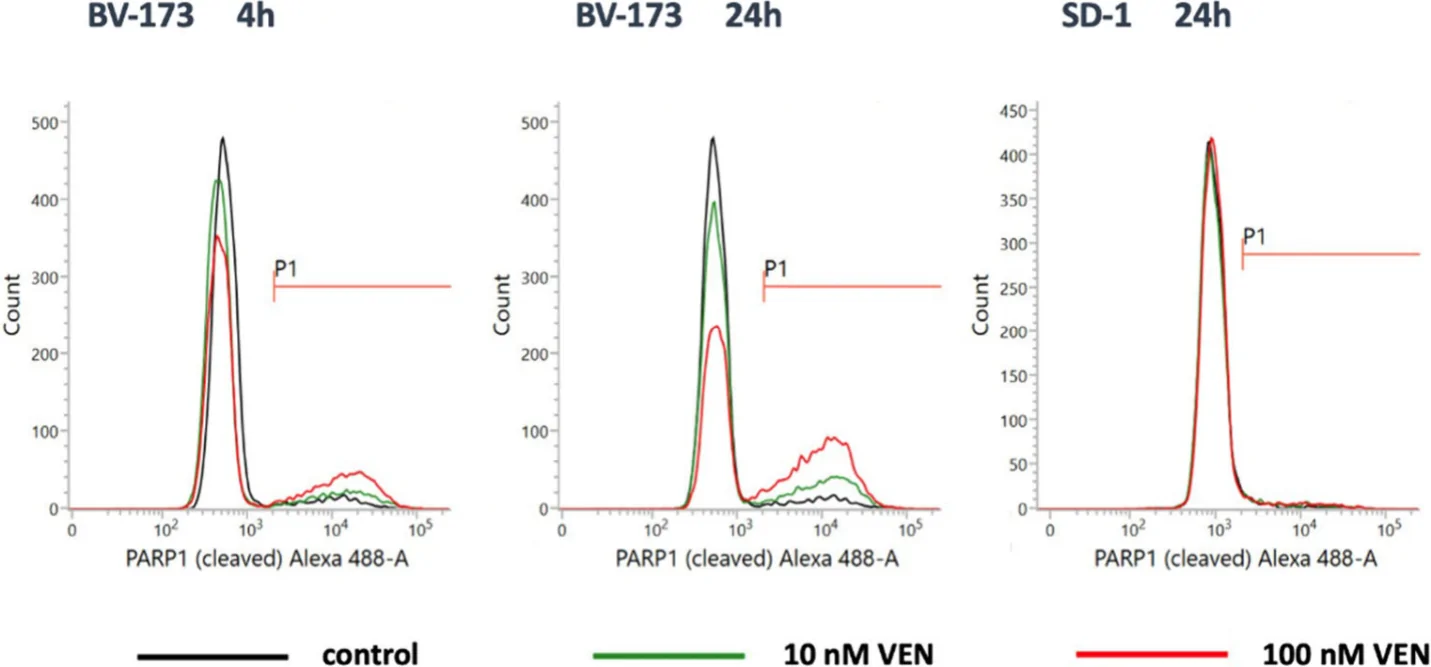
Histograms presenting the effects of exposure to VEN on the level of the cleaved PARP-1 determined by flow cytometry assay. BV-173 cells were analyzed after 4 and 24 h, whereas SD-1 cells were analyzed after 24 h; cells were treated with 10 or 100 nM VEN. Cleaved PARP was measured by flow cytometry assay in the presence of specific antibodies.

### Assessment of BCL-2 Family Protein Expression in BCR-ABL Leukemic Cell Lines

Sensitivity to VEN and the progression of apoptosis may be related to the expression of the target protein (Bcl-2) or proapoptotic and antiapoptotic proteins from the BCL-2 family. Therefore, we first evaluated the baseline expression of selected BCL-2 family proteins in nontreated BV-173 and SD-1 cells. We observed that the Bcl-2 protein is expressed at higher levels in BV-173 cells than in SD-1 cells (Figure 4). Our data indicate that BCL-2 family expression in BV-173 cells may reflect a greater association with BCL-2-related apoptotic regulation. Blocking Bcl-2 with VEN may disrupt the balance between pro- and antiapoptotic proteins, consistent with induction of apoptotic signaling. Then, we measure the expression of antiapoptotic Bcl-xl, Bcl-w, and A1Bfl1. The second critical antiapoptotic factor, Bcl-xl, also showed increased expression in BV-173 cells. The Bcl-2, Bcl-xl, and Bcl-w sequester their proapoptotic counterparts BAK and BAX and prevent their pore-forming activity.^27^ Our results showed that the three main cytoprotective proteins in the BCL-2 family are more highly expressed in sensitive cells. These observations suggest that resistance to VEN in the investigated models may not be directly associated with basal expression levels of BCL-2, BCL-w, or BCL-xl alone. On the other hand, two pro-apoptotic proteins, BAK and BID, exhibited higher expression in BV-173 cells than that in SD-1 cells. BAK, as an effector pro-apoptotic protein together with BAX, oligomerizes to form pores to induce MOMP (mitochondrial outer membrane permeabilization). The BID protein is a so-called activator of apoptosis that binds to BAK and BAX, promoting MOMP.^28^ We observed increased expression of both selected proapoptotic and antiapoptotic proteins in BV-173 cells, compared to SD-1 cells. Both cell lines expressed key members of the BCL-2 family, and the observed results reflected quantitative differences among the analyzed proteins. On the basis of the results obtained, we investigated whether the expression of BCL-2 family members is affected by VEN treatment.

**Figure 4:**
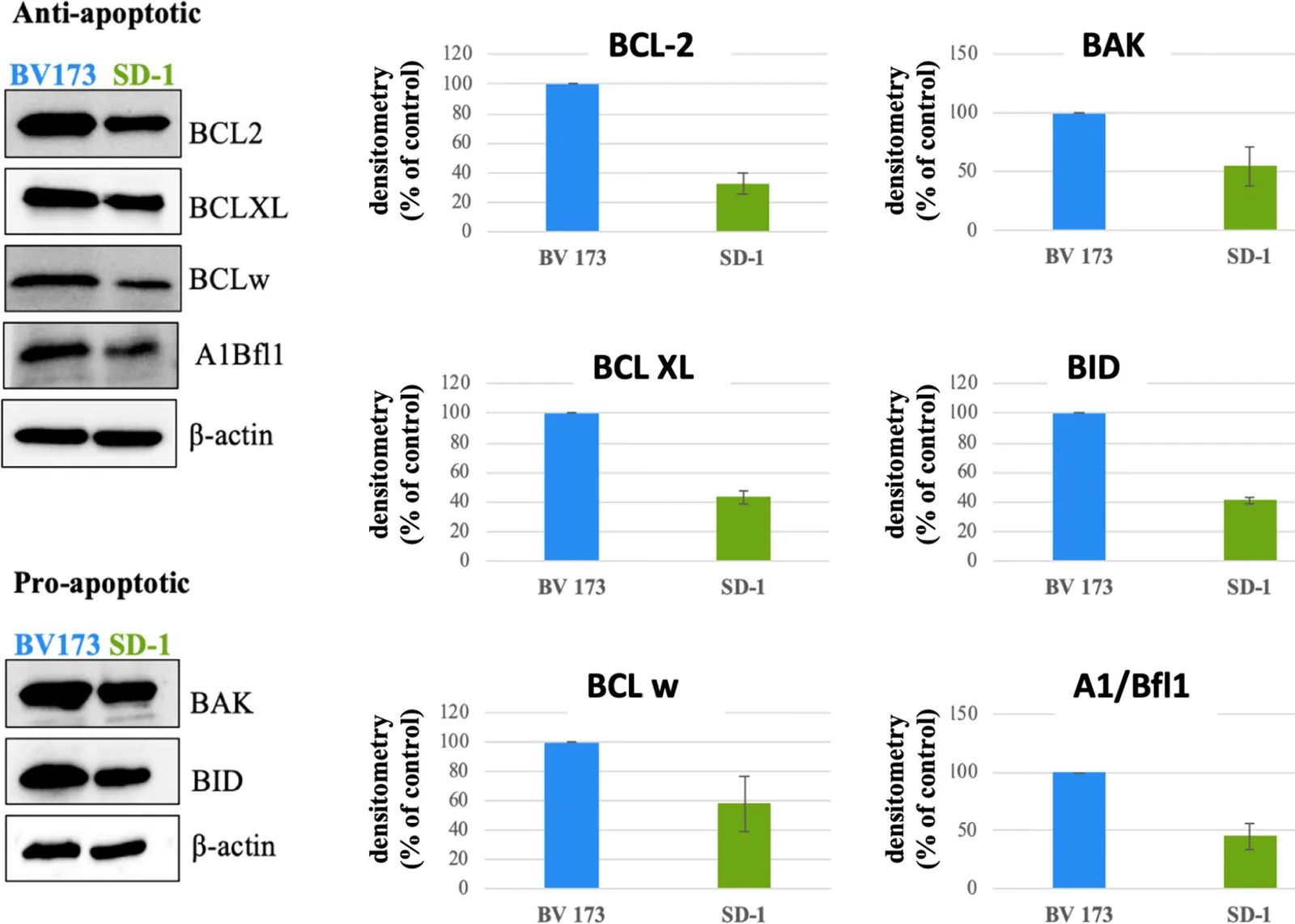
Expression of proapoptotic and antiapoptotic proteins from the BCL-2 family measured in B-ALL cell lines; SD-1 and BV-173 were used in a Western blot. The bar chart indicates protein quantification by densitometric analysis.

### Measurements of BCL-2 Family Protein Expression in BCR-ABL Leukemic Cell Lines Exposed to VEN

Because of a lack of a clear answer regarding the relationship between VEN sensitivity and BCL-2 protein family expression, we measured the expression of selected members of the BCL-2 protein family in VEN-treated cells. There was no significant effect of VEN treatment on the expression of the selected proteins. The tendency toward higher expression in more sensitive BV-173 cells remained stable and independent of the drug treatment.

### Analysis of BCL-2 Phosphorylation in VEN-Treated Philadelphia-Positive Leukemic Cell Lines

To evaluate the complex interplay between BCL-2 protein family phosphorylation and VEN, we examined BV-173 and SD-1 cell lines, which are intrinsically sensitive and resistant to VEN, respectively. We measured the phosphorylation status of the antiapoptotic proteins BCL-2 (Ser70) and Mcl-1 (Thr163), and the proapoptotic protein BAD (Ser112), after treatment with VEN. The β-actin bands were normalized to quantify band intensity, and the ratio of phosphoprotein to total protein was calculated (Figure 5). However, only the BAD protein in the BV-173 cell line showed a significant increase in the phosphorylation ratio after VEN treatment. Chong et al. reported higher S112pBAD levels in primary CLL samples from patients on VEN therapy than in paired pre-VEN patient samples.^29^ These observations may support the notion that elevated phosphorylation of BAD might be a potential treatment-associated feature observed in VEN-responsive leukemia models. Phosphorylation of BCL-2 at Ser 70 enhances the binding affinity to BAK and BIM and blocks its proapoptotic activity. Moreover, enhanced BCL-2 phosphorylation might be associated with protection against anticancer therapy.^30^ BAD phosphorylation at Ser112 is one of the phosphorylation sites that prevents binding to BCL-2 and BCL-XL. Phosphorylated BAD loses the ability to block BCL-2 and BCL-XL antiapoptotic activity.

**Figure 5:**
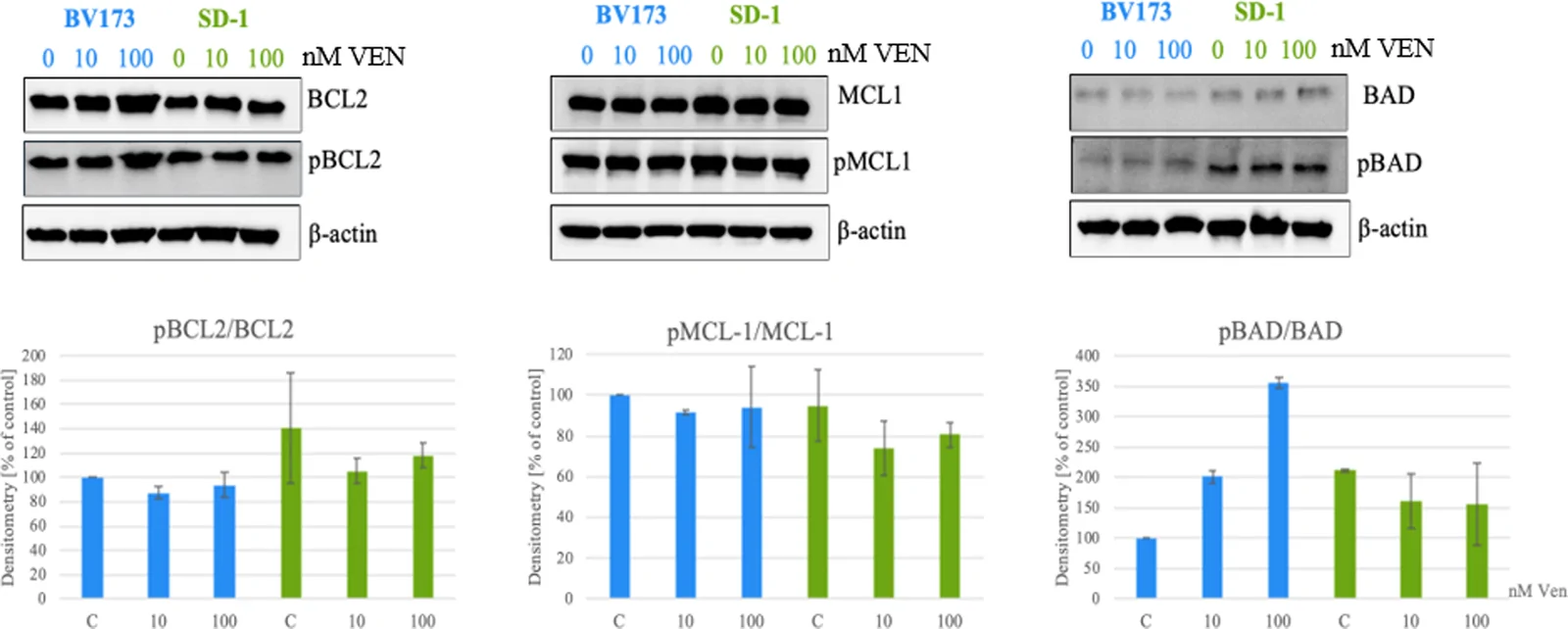
Phosphorylation of BCL-2 family protein in B-ALL cell lines exposed to VEN. Expression of S70pBCL-2, T163pMCL-1, and S112pBAD after 10 nM and 100 nM of exposure to VEN treatment for 4 h. Protein bands were quantified by Image Lab software (Bio-Rad), and values derived from phosphorylated protein were normalized by total protein. Quantitative densitometry is shown to compare phosphorylated protein expression as a ratio normalized to untreated control SD-1 cells.

### Description of the Biological Models for Raman Studies

The results described above indicate significant differences in the sensitivity to VEN between the BV-173 and SD-1 cell lines. The viability tests (MTT and PI) confirmed that BV-173 cells were more sensitive to VEN than SD-1 cells. Moreover, cleaved PARP-1 measurements indicate that, unlike in the SD-1 cell line, the drug induces apoptosis in BV-173 cells. SD-1 resistance is intrinsic and not acquired by pretreatment with the drug or by genetic modification. We showed that both cell lines expressed the VEN target protein (BCL-2) and effector and regulatory pro- and antiapoptotic proteins from the BCL-2 family, suggesting that apoptosis can be induced by VEN in both cell lines. The differences in the protein expression levels are quantitative and subtle. The differences in protein expression were cell-line-specific, and VEN did not influence protein levels. Since BV-173 and SD-1 represent the same molecular subtype of B-ALL, they are suitable models for studying differences in the VEN response within a similar phenotypic background. Therefore, we tested RS and FT-IR as alternative label-free tools to investigate biochemical markers of response to VEN. We used the same experimental conditions of VEN exposure to test whether RS and FT-IR spectroscopy could characterize metabolic changes in resistant and sensitive leukemic cell lines upon VEN treatment, thereby characterizing treatment-associated spectroscopic changes accompanying VEN-induced apoptosis.

### Baseline Spectroscopic Profiles of BV-173 and SD-1

To characterize the spectroscopic profiles of BV-173 and SD-1 cell lines carrying BCR-ABL1 fusion genes, we compared the Raman and FT-IR spectra of the control and VEN-treated cells. Raman differences specific to the BCR-ABL1 subtype to which the BV-173 and SD-1 cell lines belong were described in the work by Adamczyk et al.^23^ Here, we focused on understanding the detailed differences between these two cell lines in their responses to VEN and on potential markers of apoptosis induced by this drug. This study presents results from PLS-DA, which is the primary tool for interpreting spectroscopic data from VEN-treated B-ALL cell lines. PLS-DA is a multivariate analysis method widely used for data reduction, pattern recognition, and the development of classification models; in our study, it was primarily used to identify spectral regions responsible for the observed differences among the analyzed groups. PLS-DA models were not used as predictive tools or classifiers for diagnostic purposes but rather as exploratory methods to analyze regression vectors and evaluate the variables that contribute most to data separation. PLS-DA enabled the visualization of the spectral regions that contribute to the separation between the investigated groups. The Supporting Information includes tables that assign the Raman (Table S2) and FT-IR (Table S3) bands that have been discussed. Additionally, it presents the average Raman spectra for the studied groups and the cellular second-derivative FT-IR spectra for each sample, reinforcing the analysis of spectroscopic changes (Figures S1 and S2).

The Raman data in Figure 6A demonstrate significant differences in the biochemical background profiles between the tested cell lines. BV-173 cells exhibit unique Raman features, including distinct bands associated with increased aromatic amino acids, i.e., tyrosine and tryptophan (763 (ring breathing in Trp), 855 (ring breathing in Tyr), and 1620 cm^–1^(C = C Tyr, Trp)), as well as other proteins and lipid bands: 968, 1040, 1240, 1275, and 1656 cm^–1^. These features, along with the more intense RNA bands at 816, 915, and 1240 cm^–1^, may reflect differences in protein-associated spectral contributions, as described earlier.^23^ In contrast, SD-1 cells showed a distinct spectral profile and a set of characteristic Raman features with differences in intensity and band positions, suggesting structural differences. For SD-1 cells, PLS-DA indicates significance of two protein bands at 1450 cm^–1^ (CH_2_ deformation) and 1680 cm^–1^ (amide I), together with a number of nucleic acid bands (795 cm^–1^ (ring breathing of U, T, C), 1095 cm^–1^ (PO_2_^–^ symmetric stretching), and 1340, 1375, 1495,1585 cm^–1^) describing modes of DNA bases and it is consistent with our previous observations.^23^ Thus, there are significant differences in the baseline Raman characteristics of sensitive and resistant cells with respect to nucleic acid structure markers.

**Figure 6:**
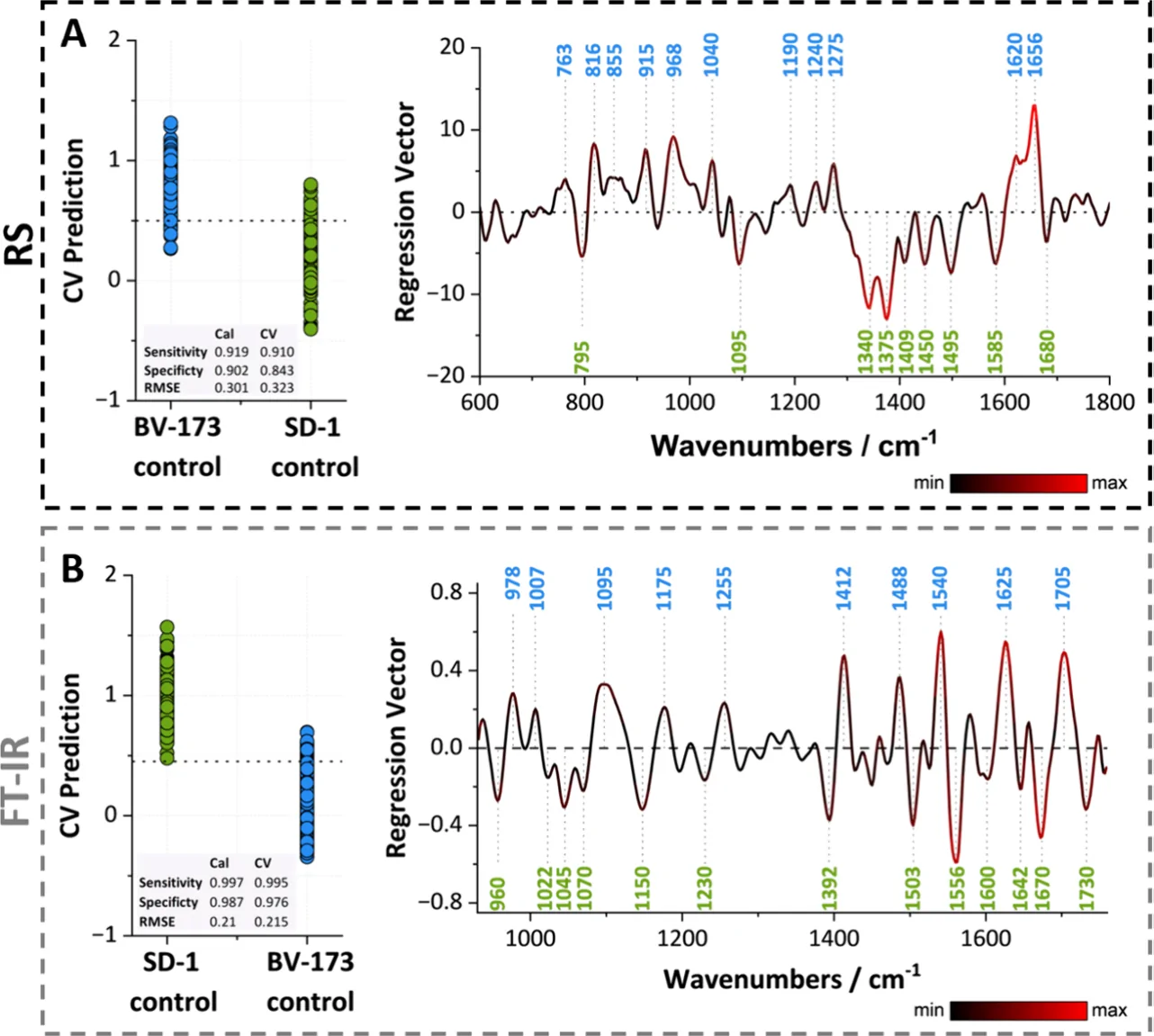
PLS-DA analysis of RS and FT-IR spectra of the SD-1 and BV-173 cells. CV results of the PLS-DA model for (A) RS and (B) FT-IR. A color-coded VIP score intensity scale has been included to indicate the bands that contribute most to the built PLS-DA model. Cal, Calibration; CV, Cross-Validation; RMSE, Root mean square error. The SD-1 cell line spectra were assigned a value of (1), and the spectra of the BV-173 cell line had a value of (0).

To verify and complement the RS data, we used FT-IR (Figure 6B) to gain better insight into protein profiles of the studied cells. Comparing the basal profiles of the examined cell lines, we identified differences in the amide II and I bands (1500–1700 cm^–1^), reflecting differences in protein secondary structure. The amide II band for BV-173 was observed at 1540 cm^–1^, while the amide I band was at 1625 cm^–1^, which may reflect differences in protein secondary structure, including possible β-sheet contributions. Other bands attributed to proteins are also present in the FT-IR spectra of BV-173 cells: 1175, 1412, and 1488 cm^–1^. For SD-1, an amide II band was present at 1556 cm^–1^, while bands at 1642 and 1670 cm^–1^ (amide I) suggested differences in protein secondary structure contributions in SD-1 corresponding to α-helices and β-turns structures, respectively. In addition, in the FT-IR spectra of SD-1 cells, bands related to carbohydrates at 1150 cm^–1^ and lipids at 1730 cm^–1^ were observed. Analysis of FT-IR spectra also revealed the importance of nucleic acid marker bands for characterizing both cell lines studied. For BV-173, we identified bands for RNA: 978, 1095, and 1255 cm^–1^ ^31–33^ and nitrogenous bases at 1705 cm^–1^,^34,35^ while for SD-1 cells, PLS-DA indicates the characteristic FT-IR bands of DNA: 960, 1022, 1045, and 1230 cm^–1^.^32,33,36^ The 1095 cm^–1^ band may also reflect a higher content of phosphorylated proteins in sensitive cells. Basal differences in nucleic acid profiles among the examined cell lines may be obscured by variations in protein secondary structure, resulting in low intensities. However, the band patterns we observed, along with their consistency with the Raman results, may reflect differences in the spectral contributions of RNA and DNA in BV-173 and SD-1. As shown in Figure 6, our study revealed clear distinctions in the baseline biochemical profiles of the cell lines analyzed, particularly in protein and nucleic acid content.

### Spectroscopic Description of VEN-Treated Cells

The analysis presented in the previous section Baseline Spectroscopic Profiles of BV-173 and SD-1 for the BV-173 and SD-1 cell lines revealed distinct spectroscopic signatures, interpreted as reflections of their different metabolic states. Such a divergence may represent an important factor influencing their response to VEN. Consequently, further analyses were conducted separately for each line with particular emphasis on spectroscopic markers of apoptosis, which may provide insight into the mechanisms underlying the observed differences.

Figure 7 shows the impact of VEN on BV-173 cells after 4 h (Figure 7A–D) and 24 h (Figure 7E–H) of drug exposure. The comparison between control cells and cells treated with 10 nM VEN for 4 h revealed the emergence of Raman bands at 728, 809, 1380, 1595, and 1695 cm^–1^, which are mainly attributed to nucleic acids. This may be associated with the initiation of apoptotic processes, as increased nucleic acid-related signals have been reported in early stages of apoptosis.^37–39^ The remaining bands at 763, 936, 988, 1030, 1120, 1245, 1306, 1360, 1420, 1660, and 1740 cm^–1^ indicate that additional changes in lipids and conformational/organizational changes in proteins accompany the early stage of apoptosis in BV-173. Tested VEN concentrations (10 nM and 100 nM) led to a decrease in the intensity of the 1010 cm^–1^ band attributed to the ring breathing modes of phenylalanine residues in proteins. This may suggest a relative reduction in protein-associated spectral contribution during the early stage of apoptosis.^38,40^ An additional increase at 763 and 1360 cm^–1^ may be associated with changes in spectral contributions from tryptophan-containing proteins. At a concentration of 100 nM, an increase in DNA (795 cm^–1^) was also observed, although these changes were dominated by stronger conformational changes in proteins (763, 988, 1030, 1120, 1253, 1360 cm^–1^). Lipid bands 1170 and 1306 cm^–1^ also play an important role. The band at 713 cm^–1^, assigned to choline-containing lipids, was also present,^40^ and their reorganization during this process may reflect membrane lipid reorganization accompanying early apoptotic processes.^38,41,42^ The BV-173 cells treated with VEN for 4 h exhibited significant FT-IR bands at 986, 1080, 1085, and 1260 cm^–1^, associated with vibrations of nucleic acids and phosphates, consistent with the Raman results. Furthermore, we observed that at the early stage of apoptosis, changes in protein secondary structure occur in the 1300–1600 cm^–1^ range, where bands from amide III, amide II, and tyrosine vibrations (1525 cm^–1^) contribute. We also indicated that, at that stage in BV-173, proteins with an α-helix structure (1642, 1667 cm^–1^) play a greater role. Both concentrations also decreased the intensity of the 1540 cm^–1^ band (amide II), relative to the control.

**Figure 7:**
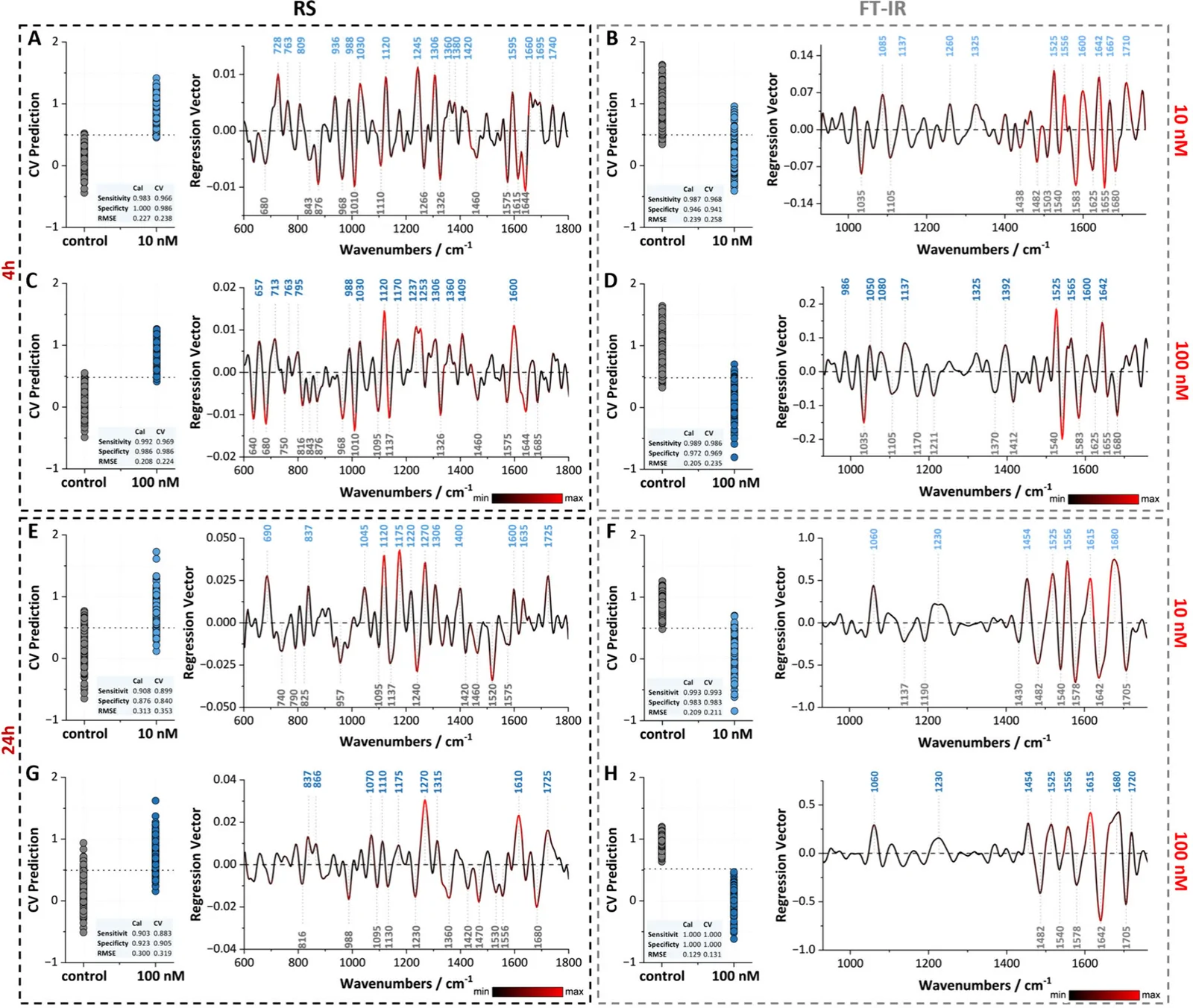
PLS-DA analysis of RS and FT-IR spectra for an in-vitro model of BCR-ABL1 cells based on the BV-173 cell line. CV results of the PLS-DA model for (A, C) RS and (B, D) FT-IR after 4 h VEN and for (E, G) RS and (F, H) FT-IR after 24 h VEN incubation for 10 nM (A, B, E, F) and 100 nM (C, D, G, H). The control spectra were assigned a value of “0” for RS and “1” for FT-IR, and the spectra of VEN-treated cells had a value of “1” for RS and “0” for FT-IR in each case. The corresponding regression vector is shown next to each PLS-DA model visualization. A color-coded VIP score intensity scale has been included to indicate the bands that contribute most to the built PLS-DA model. [Cal = Calibration; CV = Cross-Validation; RMSE = Root mean square error.]

To trace further stages of apoptosis, we analyzed BV-173 spectra treated with 10 nM and 100 nM VEN for 24 h (Figure 7E–H). The Raman assignments observed on the positive side of the regression vector shown in Figure 7E, G, which correspond to the spectra of single BV-173 cells treated with VEN, suggested that the spectra were dominated by proteins, with a prominent lipid contribution. Raman features of proteins were observed at 837, 866, 1045, 1120, 1175, 1270, 1610, and 1635 cm^–1^, and for lipids at 1070, 1175, 1270, 1306, and 1725 cm^–1^. Both concentrations after 24 h in BV-173 caused similar changes, including increases in protein and lipid content, consistent with markers of late apoptosis.^38,43,44^ Importantly, after 24 h of exposure to VEN, we observed a reduction in nucleic acid-associated Raman bands, which is consistent with previously reported spectral changes during late apoptosis.^38,43,45^ In the case of FT-IR, monitoring global changes in the secondary structure of proteins in the amide I region (1600–1700 cm^–1^) proved important for identifying a further stage of apoptosis. FT-IR spectra of cells treated with VEN for 24 h, compared with controls, showed strong bands associated with β-sheet/β-turn structures (1680 cm^–1^) and intracellular protein aggregates (1615 cm^–1^), consistent with structural alterations associated with late-stage apoptosis. VEN induces apoptosis by inhibiting BCL-2, activating caspases, and causing subsequent protein cleavage. A decrease in α-helical content and an increase in β-sheet content may be observed in late apoptotic cells due to fragmentation of peptide chains, which promotes aggregation and the formation of β-sheets.^46,47^ As observed previously, compared to 4 h, we observed the disappearance of DNA bands, which could also be linked to the progression of the apoptotic process.^46–48^

For SD-1, we also examined the impact of VEN at concentrations of 10 nM and 100 nM for 4 h (Figure 8A–D) and 24 h (Figure 8E–H). Comparison between the control and 10 nM VEN treatment after 4 h revealed changes in the Raman bands at 1040, 1137, 1170, 1245, and 1625 cm^–1^. These shifts may be associated with alterations in protein conformation or relative protein contribution. Additionally, Raman features of lipids, including fatty acids (1170 and 1306 cm^–1^), appeared. At 100 nM VEN, SD-1 cells showed new spectral features at 657, 1125, 1220, 1360, 1420, 1470, and 1610 cm^–1^, suggesting intensified modifications in the protein structure and an increase in their content (1016 cm^–1^). This response differs from that observed in the BV-173 cell line, in which a decrease in protein content was a key feature of early apoptosis. The response of SD-1 to a higher concentration also includes the appearance of lipid and fatty acid features (1080, 1306 cm^–1^), accompanied by an increase in cholesterol (701 cm^–1^).^40^ In addition to protein and lipid content modifications, shifts in the 728, 800, 1380, 1585, and 1595 cm^–1^ bands associated with nucleic acids were also observed. These results show that after only 4 h, SD-1 cells display an adaptive response to VEN rather than initiating apoptosis. The FT-IR dataset for treatment with 10 nM VEN shows bands at 960, 990, 1022, 1045, 1080, 1240, and 1137 cm^–1^. This indicates changes/increased relative contributions of nucleic-acid- and lipid-associated spectral features, consistent with DNA metabolism disorders (increased DNA content) and activation of the SD-1 cells after VEN treatment. β-turns (1670–1690 cm^–1^), being a type of irregular secondary structure, were also observed, as well as other protein bands (1392, 1454, 1495, and 1540 cm^–1^). The band at 1720 cm^–1^ indicates substantial changes in lipid composition in SD-1 cells and an increase in fatty acid content,^34^ compared to the control. These shifts may reflect early biochemical changes in DNA, proteins, and lipids, possibly related to stress responses and differences in metabolic states. In SD-1 cells treated with 100 nM VEN, in addition to an increase in the β-turns content (1690 cm^–1^), fatty acids (1720 cm^–1^), and carbohydrates (1060, 1130 cm^–1^), as in the case of a lower concentration, new FT-IR features appear at 1315, 1438, 1525, and 1660 cm^–1^. These bands are associated with lipids (1438 cm^–1^) and proteins (amide III, 1315 cm^–1^), including vibrations of tyrosine residues (1525 cm^–1^) and alterations in protein secondary structure, namely, the appearance of α-helical structures (1660 cm^–1^). Thus, once more, we observed adaptation of membrane and protein structures, reflecting the SD-1 response and suggesting treatment-associated biochemical adaptation distinct from the apoptotic profile observed in BV-173 cells.

**Figure 8:**
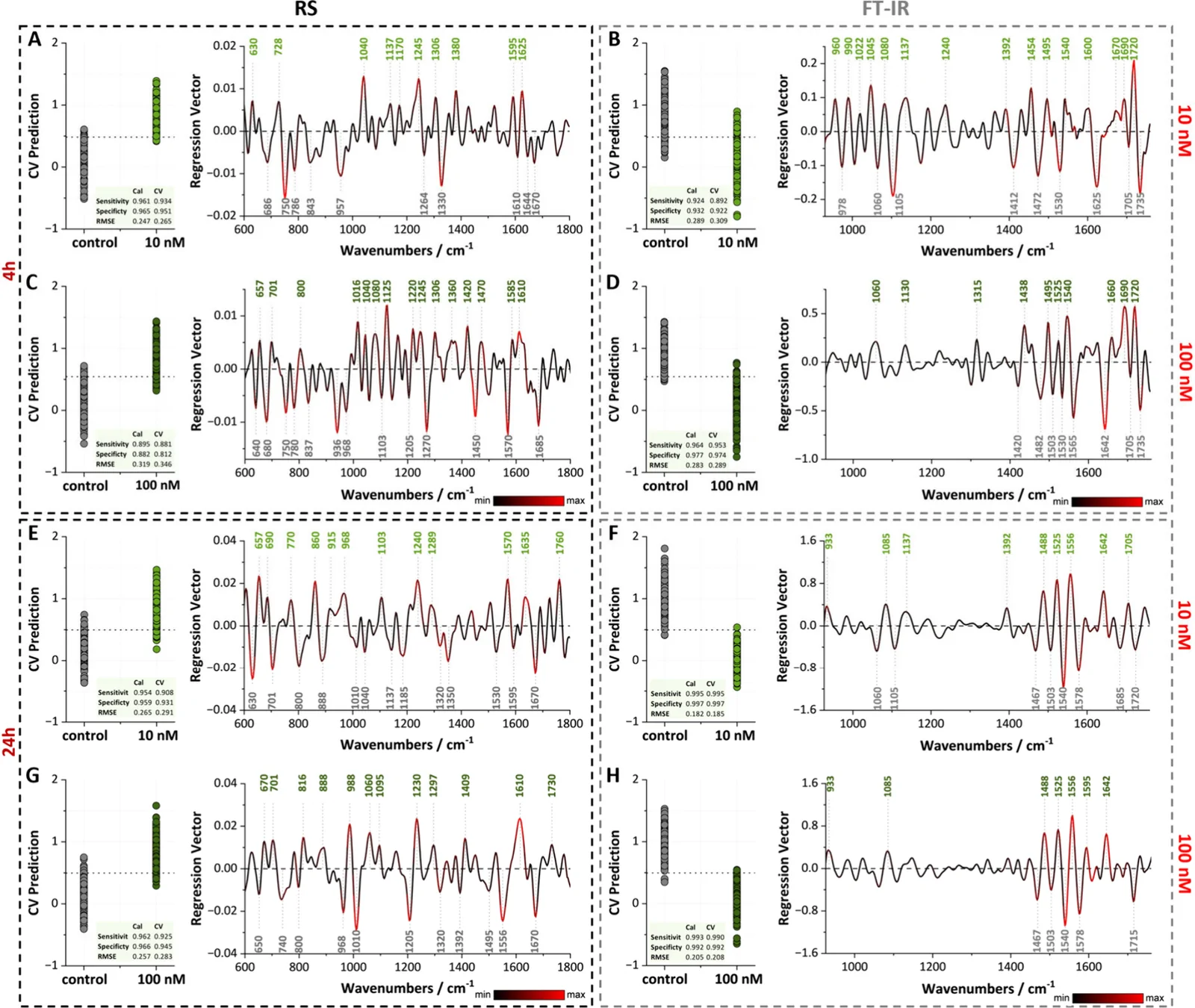
PLS-DA analysis of RS and FT-IR spectra for an in vitro model of VEN-resistance in BCR-ABL1 cells based on the SD-1 cell line. CV results of the PLS-DA model for (A, C) RS and (B, D) FT-IR after 4 h VEN and for (E, G) RS and (F, H) FT-IR after 24 h VEN incubation for 10 nM (A, B, E, F) and 100 nM (C, D, G, H). The control spectra were assigned a value of (0) for RS and (1) for FT-IR, and the spectra of VEN-treated cells had a value of “1” for RS and “0” for FT-IR in each case. The corresponding regression vector is shown next to each PLS-DA model visualization. A color-coded VIP score intensity scale has been included to indicate the bands that contribute most to the built PLS-DA model. [Cal = Calibration; CV = Cross-Validation; RMSE = Root mean square error.]

The FT-IR spectra of SD-1 cells after treatment with 10 nM VEN for 24 h, compared to the control, had several more intense bands attributed to proteins (860, 968, 1240, 1570, 1635 cm^–1^), lipids (1760 cm^–1^), and nucleic acids (770, 1289 cm^–1^). At the higher concentration (100 nM), protein bands at 988, 1060, 1230, 1297, and 1409 cm^–1^ remained contributing, as did bands indicating the presence of phenylalanine and tryptophan. Furthermore, the pronounced Raman features of lipids (1730 cm^–1^) and nucleic acids (816, 1095 cm^–1^) continued to be identified as distinctive. Similar to the findings after 4 h at 100 nM, a cholesterol band (701 cm^–1^) was evident. However, this band was characteristic of control cells at a lower concentration for both time points (4 h and 24 h). These changes reflect VEN’s effects on SD-1, resulting in cell survival. Examination of the overall response to VEN after 24 h of incubation using FT-IR showed significant changes in the amide I and II regions (1500–1700 cm^–1^). FT-IR features for 10 nM and 100 nM VEN appeared at 1392, 1488, 1525, 1556, 1595, and 1642 cm^–1^. In cells treated relative to the controls, there was a substantial shift of amide II from 1540 cm^–1^ to 1556 cm^–1^, increased tyrosine-associated spectral contribution (1525 cm^–1^), and an increase in the content of α-helical structures in proteins (1642 cm^–1^). The bands at 1085, 1705, and 933 cm^–1^, attributed to nucleic acids, were also present.^33^ The response observed in SD-1 cells was distinct from that of BV-173 cells, which exhibited protein aggregates, β-sheets, and heightened lipid content. This indicates that, after treatment with VEN, SD-1 cells changed their protein profile and nucleic acid structure. Overall, the observed spectroscopic changes in SD-1 cells may reflect treatment-associated adaptive responses distinct from the apoptotic profile identified in BV-173 cells. However, direct involvement of oxidative stress-related mechanisms or specific resistance pathways would require additional dedicated functional validation.

## Discussion

The main aim of our study was to investigate treatment-associated biochemical changes accompanying the differential response to VEN and to characterize spectroscopic features associated with VEN-induced apoptosis. In our approach, we employed a broad-spectrum spectroscopic method to provide a comprehensive view of the metabolism of the VEN-treated B-ALL cells. We applied short-term (4 h) and long-term (24 h) incubations to capture the early and late phases of apoptosis, respectively. We confirmed VEN-induced apoptosis using established flow cytometric markers (PI staining and cleaved PARP-1), thereby providing biological validation of the spectroscopic analyses. Because of the limited biological model used in our study, the identified spectral and biological differences should be interpreted as characteristic of the investigated cell models rather than a universal marker of VEN response or resistance.

In our in vitro model, we selected two B-ALL cell lines: naturally sensitive (BV-173) and naturally resistant (SD-1) to VEN. Both cell lines harbor the Philadelphia chromosome and express BCR-ABL1. We investigated the expression of several BCL-2 family proteins. Results showed a significant decrease in BCL-2 expression in SD-1 cells, compared to that in BV-173 cells. A lower level of expression of the target protein might be associated with resistance to VEN. A positive correlation between the median lethal dose of ABT-737 (a BCL-2 inhibitor similar to VEN) and the BCL-2 mRNA copy number has already been reported in cancer cell lines.^49^ The important role in response to BH3 mimetic inhibitors is played by the phosphorylation of the BCL-2 protein. Inhibiting BCL-2 phosphorylation enhances the effect of the ABT inhibitor.^50^ Upregulated BCL-2 phosphorylation at S70 reduces ROS levels and DNA damage. BCL-2 phosphorylation may respond to oxidative stress and regulate mitochondrial ROS production. S70pBCL-2 might act as an ROS sensor.^51^

The increase in cleaved PARP levels following brief exposure to VEN confirmed the induction of apoptosis in BV-173 cells, consistent with VEN’s well-documented action as a BCL-2 inhibitor in ALL models.^52–54^ We observed higher BAK protein expression in BV-173-sensitive cells than in SD-1 cells, suggesting a greater potential for BV-173 to undergo MOMP. It has previously been reported that decreased Bax and Bak expression led to an apoptosis-resistant and therefore chemotherapy-resistant phenotype.^55^ Regarding B- and T-ALL, BCL-2 expression was not correlated with a favorable response to cytotoxic drugs in vitro. However, BCL-2 levels are associated with improved cell survival under unfavorable culture conditions.^56^ The results support the importance of BCL-2 family regulation in shaping the differential treatment response in leukemia cells.

Given the potential triggers of VEN resistance, the genetic backgrounds of treated cells should be considered. The upregulated activity of the BCR-ABL1 tyrosine kinase influences the activity and expression of proteins from the BCL-2 family. BCR-ABL1-expressing cells exhibit decreased BCL-2 expression and upregulated BCL-XL expression compared with wild-type cells.^57^ BCR-ABL also regulates MCL-1 expression in CML cells, and exposure to BCR-ABL inhibitors reduces MCL-1 protein levels. Silencing of the *Mcl-1* gene is associated with increased sensitivity of BCR-ABL-positive cells to the STI571 inhibitor.^58^ The examples described lead to the conclusion that the regulation of proapoptotic and antiapoptotic proteins of the BCL-2 family cannot be considered without the influence of BCR-ABL on cell signaling. Even though BV-173 and SD-1 cells are BCR-ABL-positive, they differ in response to VEN treatment. Since we did not find many significant differences in the expression of the BCL-2 protein family, we therefore investigated metabolic differences using spectroscopic methods.

First, we compared untreated cells to identify cell-line-specific differences between the two cell lines examined. Characterization of the baseline spectroscopic differences provided insights into their biochemical and metabolic characteristics. Both Raman and FT-IR spectra indicated that BV-173 cells have more ordered protein secondary structures than do SD-1 cells. The SD-1 cell line exhibits a diverse protein profile with a mix of secondary structures. These differences in SD-1 indicated distinct protein conformations compared to BV-173 and may reflect alternative protein folding or protein–protein interactions.^48,59^ In the BV-173 cell line, we also observed spectra characteristic of aromatic amino acids, which may be related to post-translational modifications and signaling pathways present in this cell. FT-IR analysis revealed carbohydrate bands in the SD-1 spectra, suggesting a significant presence of polysaccharides and oligosaccharides. The increased carbohydrate content may be related to various cellular functions associated with lipid and protein modifications. Carbohydrates also modify cell function by forming a glycocalyx around the cell membrane.^60^ Our results also indicate the difference in the spectral characteristics of nucleic acids. BV-173 shows a greater RNA—associated spectral contribution compared with SD-1, where DNA appears to be the most contributing. The characteristics of the SD-1 nucleic acid profile were consistent with those reported in our previous study.^23^ Although no direct evidence links total cellular RNA levels to BCL-2 expression in this model, previous reports indicate that BCL-2 overexpression in resistant leukemia cells may involve mRNA stabilization mechanisms, including nucleolin-mediated regulation.^61^ The differences in RNA spectral features between BV-173 and SD-1 may indicate broader transcriptional or post-transcriptional changes linked to apoptosis. Vibrational spectroscopy captures overall biochemical shifts rather than those of gene-specific expression. Since substantial baseline biochemical differences existed between the investigated cell lines, subsequent analyses focused primarily on within-cell-line comparisons between untreated and VEN-treated conditions.

Biochemical changes observed in response to different VEN concentrations and varying incubation times highlight differences in drug response and sensitivity mechanisms in BV-173, shedding light on the biochemical pathways underlying the cellular response to the drug. Prominent spectroscopic changes occurred after 4 h of exposure to VEN (increase in DNA/RNA signals in RS at 728, 809, and 1380 cm^–1^, a decrease in protein content at 1010 cm^–1^, the appearance of choline-rich lipids at 713 cm^–1^), and were consistent with spectroscopic features previously associated with early apoptotic response. In response to apoptotic signals, the cell initiates a cascade of events in which chromatin gradually condenses and the nucleus fragments. The relative increase in nucleic-acid-associated signals may reflect chromatin condensation and changes in cellular composition rather than an increase in DNA amount. At the same time, a reduction in the lipid pool is observed, associated with the reorganization of the cell membrane–cell shrinkage, and exposure of phosphatidylserine on the outer membrane surface.^38,62^ After 24 h, signals associated with advanced protein degradation (β-sheet in FT-IR at 1615 and 1680 cm^–1^), increased phospholipid content, and DNA loss dominate, consistent with progression toward late apoptotic stages.^43,47^ The presented changes were more pronounced at 100 nM and accelerate lipid reorganization and protein aggregation. The above conclusions correspond to cleaved PARP-1 and PI staining results, which show changes visible after 4 h and intensifying after 24 h of incubation with VEN.

In contrast, SD-1 displayed a treatment-associated response profile distinct from the apoptotic pattern observed in BV-173 cells, maintaining the structural integrity of proteins, lipids, and nucleic acids even at higher drug concentrations. After 4 h of SD-1 exposure to VEN, spectroscopic changes were visible and intensified after 24 h, suggesting a treatment-associated response distinct from the apoptotic profile observed in BV-173 cells. Increased contribution from protein signals (RS: 1016 cm^–1^), including aromatic amino acids (RS: 1360, 1625, 1610 cm^–1^; FT-IR: 1525 cm^–1^) and the appearance of α-helical structures (1660, 1642 cm^–1^) may reflect treatment-associated alterations in protein conformation and biochemical organization. It has previously been reported that the biosynthesis of tyrosine and tryptophan, along with the biosynthesis and metabolism of phenylalanine, were involved in the chemoresistance of the K562 leukemic cell line.^16^ Preservation of lipids also appears crucial. In SD-1, an increase in lipid signals, including fatty acids (RS: 1306, 1730, 1760 cm^–1^), phospholipids and their esters (RS: 1080, 1170 cm^–1^; FT-IR: 1720 cm^–1^), may indicate alterations in membrane-associated lipid composition during VEN exposure. The involvement of fatty acids seems to be more significant at short incubation times, whereas after 24 h, the involvement of lipid esters is observed. Given previous reports on the importance of cholesterol metabolism in promoting drug resistance,^16,63^ the band at 701 cm^–1^, commonly attributed to cholesterol-related vibrations, showed increased contribution in SD-1 cells after treatment with 100 nM VEN. It is known that dysregulation of cholesterol metabolism significantly impacts leukemia cell function and drug resistance, among other things, by enhancing proliferation and stress adaptation.^64,65^ Although SD-1 showed an increase in nucleic acid signals, the vibrations of the nucleic acid skeleton remained unchanged, suggesting relative preservation of nucleic acid structural integrity in SD-1 cells during VEN exposure. It should be emphasized that the vibrational spectra of intact cells represent highly complex, overlapping biochemical signals. Individual Raman or FT-IR bands cannot be attributed exclusively to a single molecular species or specific biochemical pathway, as many biomolecules contribute to similar spectral regions. Our interpretations are based on comparative analysis (control vs treated; sensitive vs resistant), consistency across two complementary vibrational modalities, and regression-vector patterns derived from multivariate models, rather than on isolated-band attribution. Nevertheless, alternative biochemical contributions within the same spectral ranges cannot be excluded. Further orthogonal validation (e.g., targeted lipidomics, transcriptomic, or proteomic profiling) would be required to confirm the precise molecular mechanisms underlying the observed spectral differences. Notably, the principal spectral regions identified in the regression vectors were reproducibly observed across normalization strategies and replicate combinations, supporting their internal robustness within the experimental framework studied (Figures S6–S10).

Several limitations of the present study should be acknowledged. First, the study was performed using only two BCP-ALL cell lines representing distinct VEN-sensitive and VEN-resistant phenotypes, which limits generalization of the presented findings. Second, substantial replicate-associated spectral variability was observed across independent biological experiments, necessitating additional EMSC-based harmonization before chemometric analysis (Figures S11—S15). Although this approach improved the robustness of treatment-associated spectral trends, it also influenced the global variance structure of the data set and may partially reduce replicate-associated biological variability alongside technical variability. Third, the presented PLS-DA models were designed primarily for the exploratory characterization of treatment-associated spectral patterns rather than for the development of clinically generalizable predictive classifiers. Finally, Raman and FT-IR spectral assignments are not uniquely specific to individual biomolecules or molecular pathways and therefore should be interpreted cautiously in the context of complex cellular systems.

## Conclusions

Our integrative approach based on spectroscopy and protein profiling revealed distinct apoptotic and biochemical responses to VEN in the B-ALL cell lines. Comparative analysis of VEN-sensitive and VEN-resistant B-ALL cell lines revealed distinct metabolic and structural characteristics associated with the differential VEN response. Observed differences in lipid-, protein-, and nucleic-acid-associated spectral features may contribute to understanding treatment-associated cellular responses to VEN. However, due to the limited biological model used in this study, the present findings should be interpreted with caution as proof-of-concept observations in a controlled cell-line model. Extension of this approach to additional leukemia models and primary patient-derived samples is necessary to validate its broader applicability. Further research on the functional significance of the observed spectral signatures may contribute to a better understanding of the mechanisms of the cell response to treatment and the processes involved in the development of therapeutic resistance. The results indicate that cell-line-specific biochemical and structural features may influence the diversity of drug-response profiles in leukemia.

## Supplementary Material



## Notes

Materials and methods: average Raman spectra (Figure S1) and average FT-IR spectra in the second derivative (Figure S2); number of Raman and FT-IR spectra used in chemometric analyses (Table S1); permutation test (Figures S3–S5); heat maps of VIP values for PLS-DA models in different normalization variants and datasets (Figures S6–S10); assignments of Raman (Table S2) and FT-IR (Table S3) bands observed in the spectra of the tested samples; PLS-DA results from independent replicate-level validation for RS and FT-IR data (S11–S15) (PDF)
