# Supplementary material for: Spectroscopic Signature of Apoptosis Induced by Venetoclax in Philadelphia-Positive Acute Lymphoblastic Leukemia
Source: Anal Chem. 2026 Aug 14;98(33):23998–4011. doi: 10.1021/acs.analchem.5c07689 (PMC13523549; doi:10.1021/acs.analchem.5c07689)
Supplement: Supplementary file 1 [file ac5c07689_si_001.pdf]

Spectroscopic signature of apoptosis induced by Venetoclax in *Philadelphia*-positive acute lymphoblastic leukemia

Marta Szewczyk[1#], Patrycja Dawiec[2,3#], Anna M. Nowakowska[2], Justyna Jakubowska[1], Agata Pastorczak[4], Małgorzata Barańska[2], Wojciech Młynarski[1], Katarzyna Majzner[2*], Kinga Ostrowska[1*]

[1]Medical University of Lodz, Department of Pediatrics, Oncology and Hematology, Pomorska 251, 92-513 Lodz, Poland

[2]Jagiellonian University, Faculty of Chemistry, Department of Chemical Physics, Gronostajowa 2, 30-387 Krakow, Poland

[3]Jagiellonian University, Doctoral School of Exact and Natural Sciences, Lojasiewicza 11, 30-348 Krakow, Poland

[4]Medical University of Lodz, Department of Genetic Predisposition to Cancer, Czechoslowacka 4, 92-216 Lodz, Poland

[#] Equal contribution

* Corresponding Authors.

E-mail addresses: kinga.ostrowska@umed.lodz.pl; katarzyna.b.majzner@uj.edu.pl

Contents

1. **Materials and methods……..……………………………………..…..………..………2**
2. **Spectral analysis of VEN-treated cells……..…………………………………...………6**
3. **Band assignment tables……..……………………….…………………………...…14**
4. **Independent replicate-level validation...……………………...……………………..15**
5. **References …………………………………………………...………………..…21**

1. Materials and methods

1.1. Cell Cytotoxicity

The cytostatic/cytotoxic effects of VEN (ABT-199) (VEN; Selleckchem) in ALL cell lines were tested using 3-(4,5-dimethylthiazol-2-yl)-2,5-diphenyltetrazolium bromide (MTT) assay. For experiments, cells were seeded onto a 96-well plate at a density of 0.2 × 10^6/ml for SD-1, 0.3 × 10^6/ml for BV-173, and 0.5 × 10^6/ml for SupB15 and RS4;11 in 100 µL culture medium containing escalating concentrations of VEN (0.005-2 µM). Non-treated cells were cultivated as a control. After 20 h of incubation, 10 µl of MTT solution (5 mg/ml) was added to each well and incubated for another 4 h. After the 24 h incubation period, 150 µL of acidified isopropyl alcohol (0.04 N HCl) was added to each well to solubilize the formazan crystals. Absorbance was measured at 560 nm using an MPR-96 visible microplate reader (Dynamica, Livingstone, UK). Based on the results from 3 independent experiments, IC50 values were calculated.

1.2. Propidium iodide staining

BV-173 and SD-1 cells were treated with 10 and 100 nM VEN for 4 h and 24 h. At the indicated time, 0.1 × 10^6 cells were collected, washed with ice-cold PBS and resuspended in 100 µl of PBS containing 5 µL of propidium iodide solution (Becton Dickinson, Franklin Lakes, NJ, USA). After 15 min of incubation (room temperature, darkness), 300 µL of PBS was added to each sample, and cells were acquired on a FACSLyric cytometer using BD FACSuite™ software (Becton Dickinson, Franklin Lakes, NJ, USA).

1.3. Detection of cleaved PARP1

After 4 h and 24 h of incubation with VEN, 0.7 × 10^6/ml cells were harvested and fixed with Fixation Buffer (Becton Dickinson, Franklin Lakes, NJ, USA) for 15 min at 37°C. After fixation, the cells were permeabilized with ice-cold BD Phosflow™ Perm Buffer III (Becton Dickinson, Franklin Lakes, NJ, USA) for 30 min on ice. After the washing step with staining buffer (PBS + 2% FBS), cells were incubated with AF488-conjugated anti-cleaved PARP1 antibody (eBioscience 53666842) in a final volume of 100 µL of staining buffer for 30 min in the dark. Following incubation, samples were washed with staining buffer and acquired on a FACSLyric cytometer using BD FACSuite™ software (Becton Dickinson, Franklin Lakes, NJ, USA).

1.4. Preparing cells for RS/ FT-IR measurements

After 4 h and 24 h of incubation with VEN, 5×10^6 BV-173 and SD-1 cells were harvested and centrifuged. After the washing step with PBS (without Ca^{2+} and Mg^{2+}), cells were fixed with 0.5% glutaraldehyde (Merck, G7651) for 10 min at room temperature, then washed three times with PBS (without Ca^{2+} and Mg^{2+}) to remove the excess amount of fixative. Then, the cells were suspended in PBS and stored at 4°C until Raman measurements.

1.5. RS measurements

Single-cell Raman images were acquired with a WITec Alpha 300 confocal Raman microscope (WITec GmbH, Ulm, Germany) equipped with an air-cooled laser of 532 nm excitation wavelength and a CCD detector (Andor Technology Ltd., Belfast, Northern Ireland). Cells suspended in saline buffer (200-500 µL) were placed on CaF_2 windows (Crystran LTD, Poole, UK, Raman grade). The measurements were conducted using a Zeiss W Plan-Apochromat water-dipping objective (NA=1; Oberkochen, Germany). The cells were left to settle and immobilize for at least 30 minutes after sample deposition. Only undamaged oval-shaped cells without visible abnormalities were measured, with a sampling density of 1 µm, an integration time of 0.5 s per spectrum, and a laser power of 50 mW (measured before the objective using the microscope's integrated power meter). All measurements were performed on fixed

cells immersed in buffer, which ensured efficient heat dissipation during acquisition. No signs of photothermal damage or spectral instability were observed under these conditions. Three independent biological replicates were measured for each BCP-ALL cell line. At least 50 cells from different areas of each sample were measured and analyzed.

1.6. FT-IR measurements

The BCP-ALL cells were washed with doubly deionized water to remove residual PBS and left to air-dry prior to FT-IR measurements. The experiments were performed using the Varian 620-IR microscope coupled to a 670-IR spectrometer, equipped with a 128×128-pixel focal plane array (FPA) detector, providing a spatial resolution of 5.5 µm per pixel in transmission mode. Measurements were performed with a 15× Cassegrainian objective and condenser, collecting 128 and 64 co-scans for background and sample images, respectively (standard resolution). All spectra were recorded in the 900-3600 cm^{-1} region with a spectral resolution of 4 cm^{-1}. For each sample, three IR images, each 700 µm × 700 µm, were collected. Measurements were made for three independent biological experiments.

1.7. Spectral Data Post-Processing and analysis of spectroscopic data

Raman spectral data post-processing consisted of the removal of artifacts from cosmic rays (cosmic ray removal, filter size: 3, and dynamic factor: 8) and background correction using the 3rd-order polynomial fitting, which was conducted using Project FIVE 5.3 Plus software (WITec GmbH, Germany). Then, k-means cluster analysis (KMCA) was performed to obtain a single-cell average spectrum of each measured cell (whole-cell cluster), as presented in the scheme below:

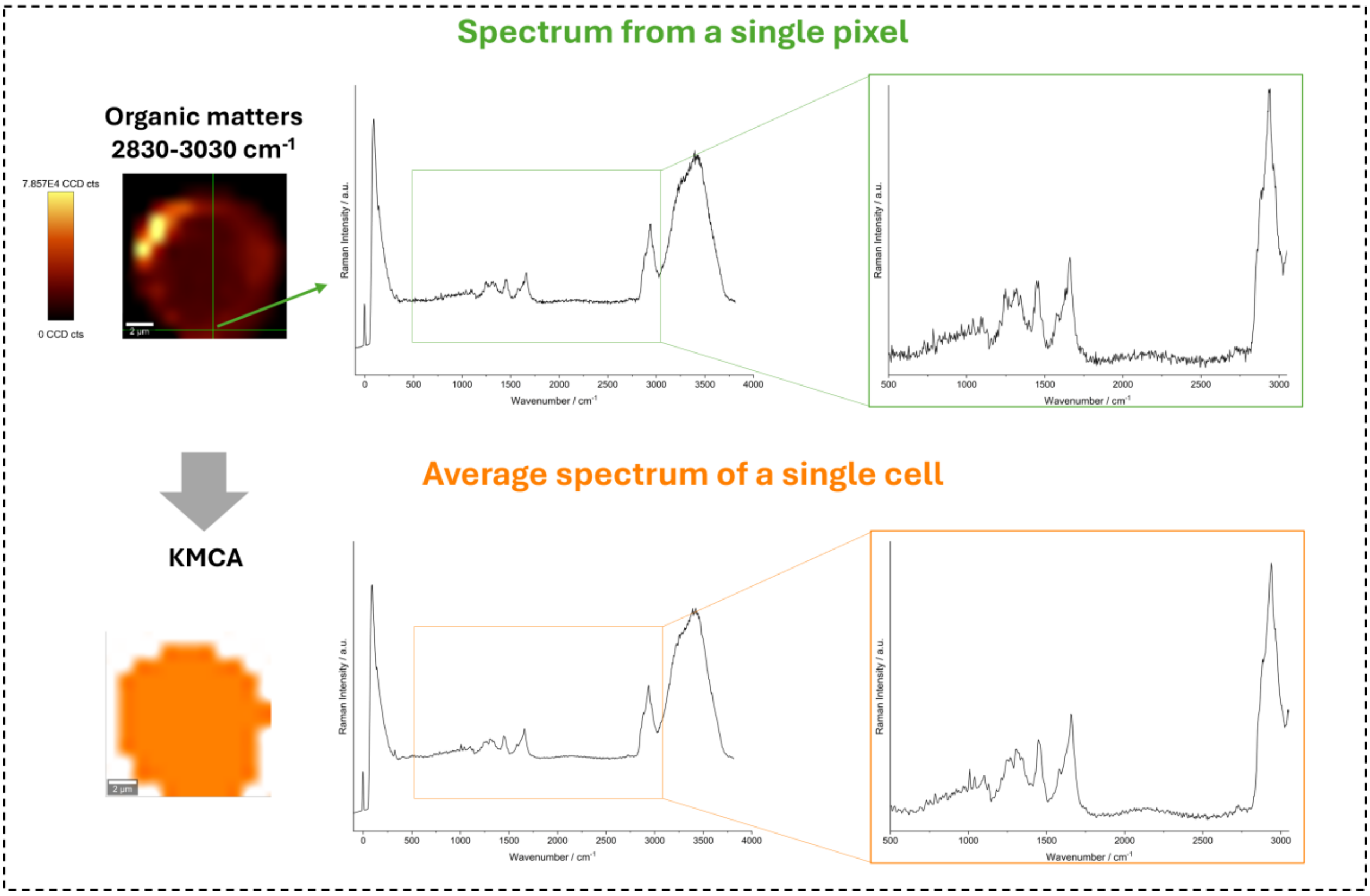

KMCA segmentation was performed using spectral similarity to separate cellular and background regions, and the whole-cell cluster was subsequently used for the calculation of average single-cell spectra used in chemometric analyses.

Infrared data were initially analyzed using CytoSpec software (ver. 2.00.07). First, we applied the quality test (sample thickness criterion) to exclude spectra from the cell-free area of the CaF_2 window from further analysis. This operation was performed in the range of 1620-1680 cm^{-1}. Next, PCA-based noise reduction with 15 PCs was applied to eliminate spectral noise, and then the spectra were smoothed using a Savitzky-Golay filter (13 points). The number of PCs used for PCA-based noise reduction was empirically selected to preserve major spectral features while minimizing high-frequency noise. Using a self-written algorithm in Python, smaller cellular regions were extracted from the IR images, for which average spectra were calculated. Up to 300 average spectra were extracted for each experimental condition (100 per biological replicate); spectra not meeting the quality-control criteria were subsequently excluded.

Chemometric analysis was performed using PLS-Toolbox Solo Mia software (Eigenvector Research, Mason, USA). Before conducting the Partial Least Squares Discriminant Analysis (PLS-DA) on Raman spectra, preprocessing involved smoothing (Savitzky-Golay, polynomial degree 3, 13 points), EMSC, and Mean Centering. For FT-IR spectra, transformations included the second derivative (also with Savitzky-Golay, polynomial 3, and 17 points), along with MSC and Mean Centering. The same preprocessing strategy was applied consistently to all FT-IR datasets to improve spectral comparability and reduce high-frequency noise inherent in FPA-based measurements. PLS-DA was performed in the spectral range of 600-1800 cm^{-1} (RS) and 926-1760 cm^{-1} (FT-IR). Preprocessing parameters were applied consistently across all datasets and were not optimized based on model performance.

Initial PCA analysis performed after conventional sequential preprocessing (cosmic ray removal, baseline correction, Savitzky–Golay smoothing, vector normalization, and mean centering) revealed dominant replicate-associated spectral variability, primarily reflected by baseline-related contributions in the PC1 loading profiles. Since conventional preprocessing applied independently to each spectrum was insufficient to homogenize spectra acquired across independent biological replicates and measurement sessions, an additional EMSC-based preprocessing strategy was applied prior to chemometric analysis.

EMSC preprocessing was performed jointly on the complete Raman dataset using the mean spectrum of the dataset as the reference spectrum. In this approach, EMSC was used not only for normalization but also as a data-driven harmonization strategy intended to reduce dominant replicate-associated spectral variability prior to chemometric analysis. The applied approach was inspired by previously published EMSC-based harmonization workflows developed for vibrational spectroscopy datasets acquired under varying experimental and instrumental conditions.[1–3]

For each PLS-DA model, cross-validation was performed using the Venetian blinds method with 10 data splits based on the spectral index in the data matrix (samples per blind = 10). Spectra were systematically assigned such that every tenth observation was allocated to the same fold. In each iteration, approximately 10% of the spectra constituted the validation subset, and each spectrum served once as validation data across the full cross-validation cycle. Cross-validation was conducted at the level of single-cell average spectra to evaluate internal model consistency within the complete dataset. Outlier detection was performed using Q-residuals and Hotelling's T^2 statistics, with default thresholds implemented in PLS-Toolbox. Model performance was assessed using sensitivity, specificity, and root-mean-square error (RMSE) for both calibration and cross-validation.

To evaluate whether the observed class separation exceeded chance-level expectations, permutation testing was performed with 200 permutations per model. For each permutation, class labels were randomly reassigned while preserving the data structure. Plots of the Standardized Sum of Squares Y versus correlation are presented in Figs. S3–S5.

To assess the robustness of discriminative spectral regions, additional analyses were performed: (i) alternative preprocessing strategies (EMSC, MSC, SNV), and (ii) models constructed using pairwise combinations of biological replicates. The resulting variable importance in projection (VIP) values were

visualized as heatmaps (Figs. S6–S10) to evaluate the consistency of spectral contributions across normalization methods and replicate configurations.

Regression vectors were examined alongside VIP scores to identify the spectral regions that contributed most strongly to group differentiation. As the models were designed for exploratory interpretation of treatment-associated spectral variance rather than predictive deployment, no parameter tuning based on validation accuracy was performed.

Average Raman spectra for each experimental condition were calculated by averaging single-cell spectra (whole-cell clusters) used in PLS-DA (Table S1). The same procedure was applied to second-derivative FT-IR spectra. Preprocessing steps were identical to those used in chemometric modeling. The number of spectra contributing to each group average is provided in Table S1. Standard deviations were calculated at the single-cell level. Data visualization was performed with OriginPro 2024b (OriginLab, Northampton, MA, USA).

1.8. Workflow of spectral preprocessing and validation strategy

To evaluate the robustness of treatment-associated spectral trends and address potential information leakage associated with single-cell cross-validation, additional leave-one-replicate-out validation was performed as an independent replicate-level validation strategy. Models were constructed using two biological replicates and tested on the third independent replicate. Model robustness was additionally assessed using permutation testing, comparison of regression vectors, and comparison of VIP heatmaps between validation strategies. These analyses were used to evaluate the consistency of discriminative spectral regions across validation approaches. We acknowledge that EMSC-based harmonization influences the global variance structure of the dataset and may partially reduce replicate-associated biological variability together with technical variability. Therefore, the presented models should be interpreted primarily as exploratory tools for characterizing treatment-associated spectral trends rather than as fully generalizable predictive classifiers.

2. Spectral analysis of VEN-treated cells

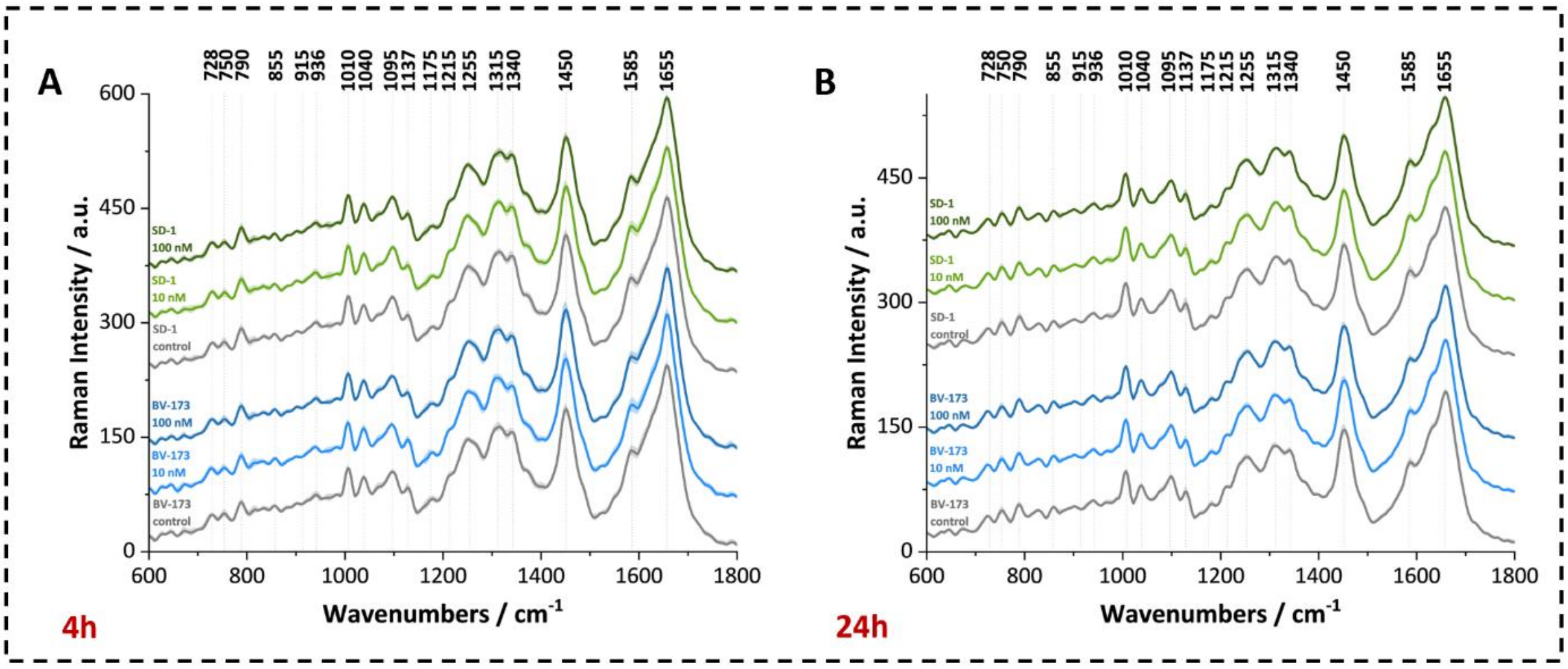

Fig. S1 Average Raman spectra of the whole cell area for BV-173 and SD-1 after 4 h (A) and 24 h (B) incubation with VEN in the regions of 600-1800 cm^{-1}. Shading denotes ± SD.

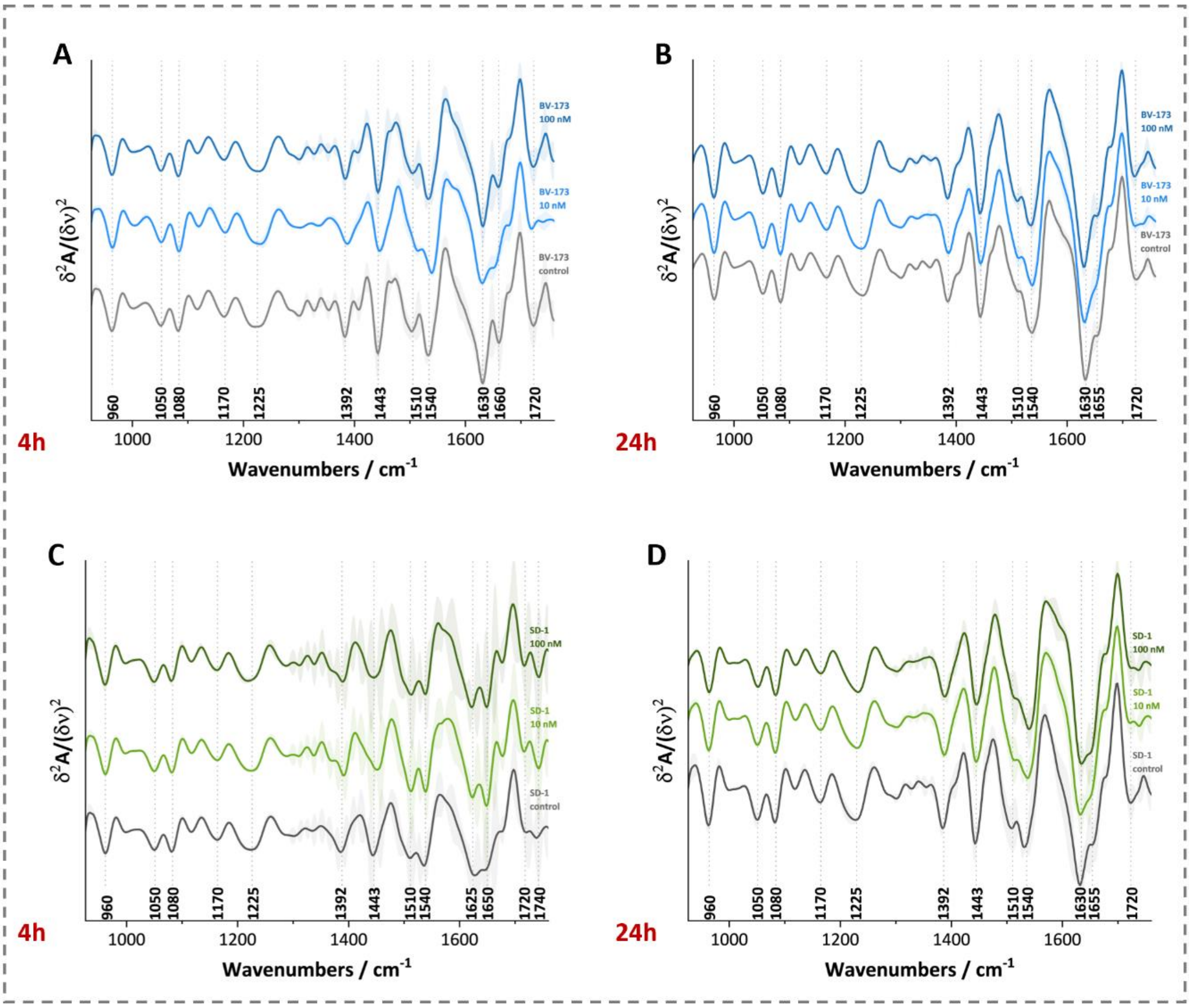

Fig. S2 Average second derivative FT-IR spectra of BV-173 for 4 h (A) and 24 h (B) time incubation with VEN and SD-1 for 4 h (C) and 24 h (D) time incubation with VEN in the regions of 926-1760 cm^{-1}. Shading denotes ± SD.

Tab. S1 The number of Raman and FT-IR spectra used in chemometric analyses. Differences in the number of spectra between groups resulted from the exclusion of low-quality or non-cellular spectra during modality-specific quality-control and spectral extraction procedures.

	BV-173			SD-1		
**Sample / Time**	**control**	**10 nM**	**100 nM**	**control**	**10 nM**	**100 nM**
			**Raman**			
**4 h**	169	174	131	144	152	143
**24 h**	140	153	154	145	174	159
			**FT-IR**			
**4 h**	296	291	296	284	292	284
**24 h**	298	283	277	284	292	296

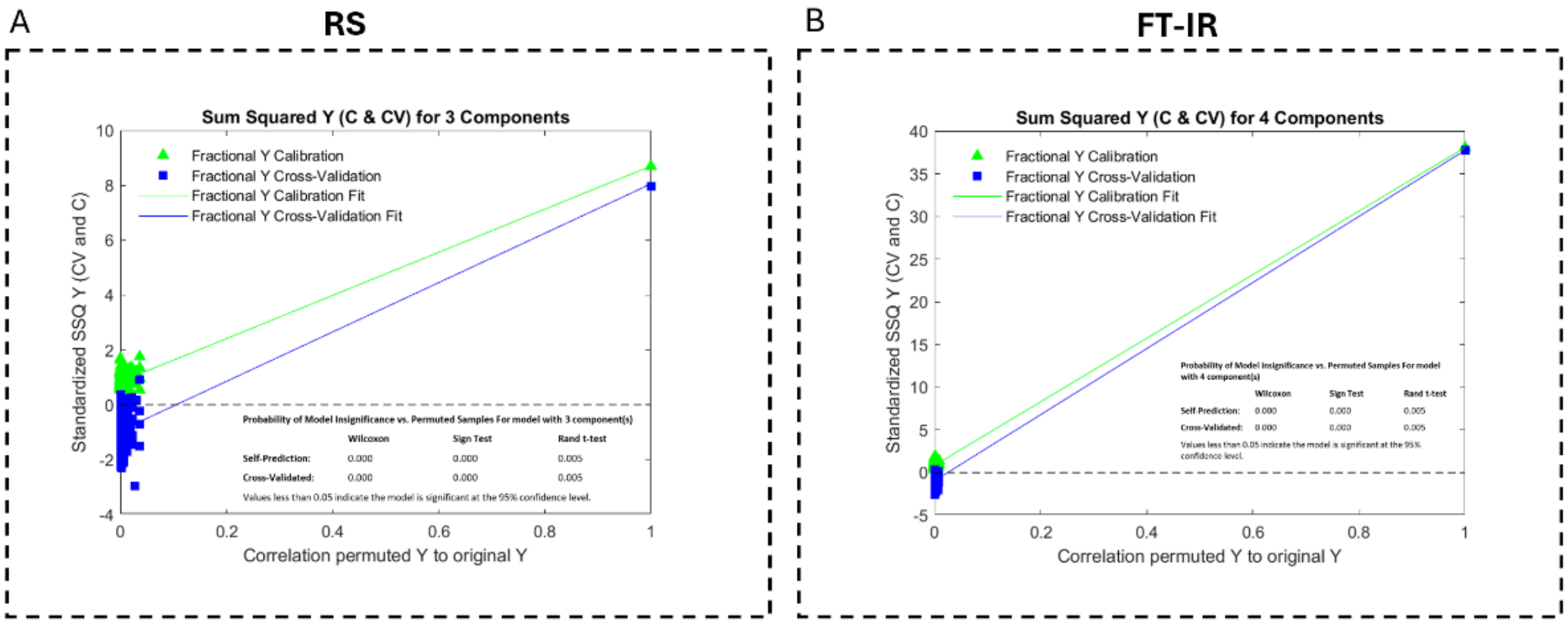

Fig. S3 Permutation test for PLS-DA of RS (A) and FT-IR (B) spectra of the SD-1 and BV-173 cells. Plot of standardized Sum Square Y vs. correlation for 200 permutations. The tables display the probability of the model insignificance. Values less than 0.05 indicate the model is significant at the 95% confidence level. The presented values indicate that the observed separation exceeds chance-level expectations.

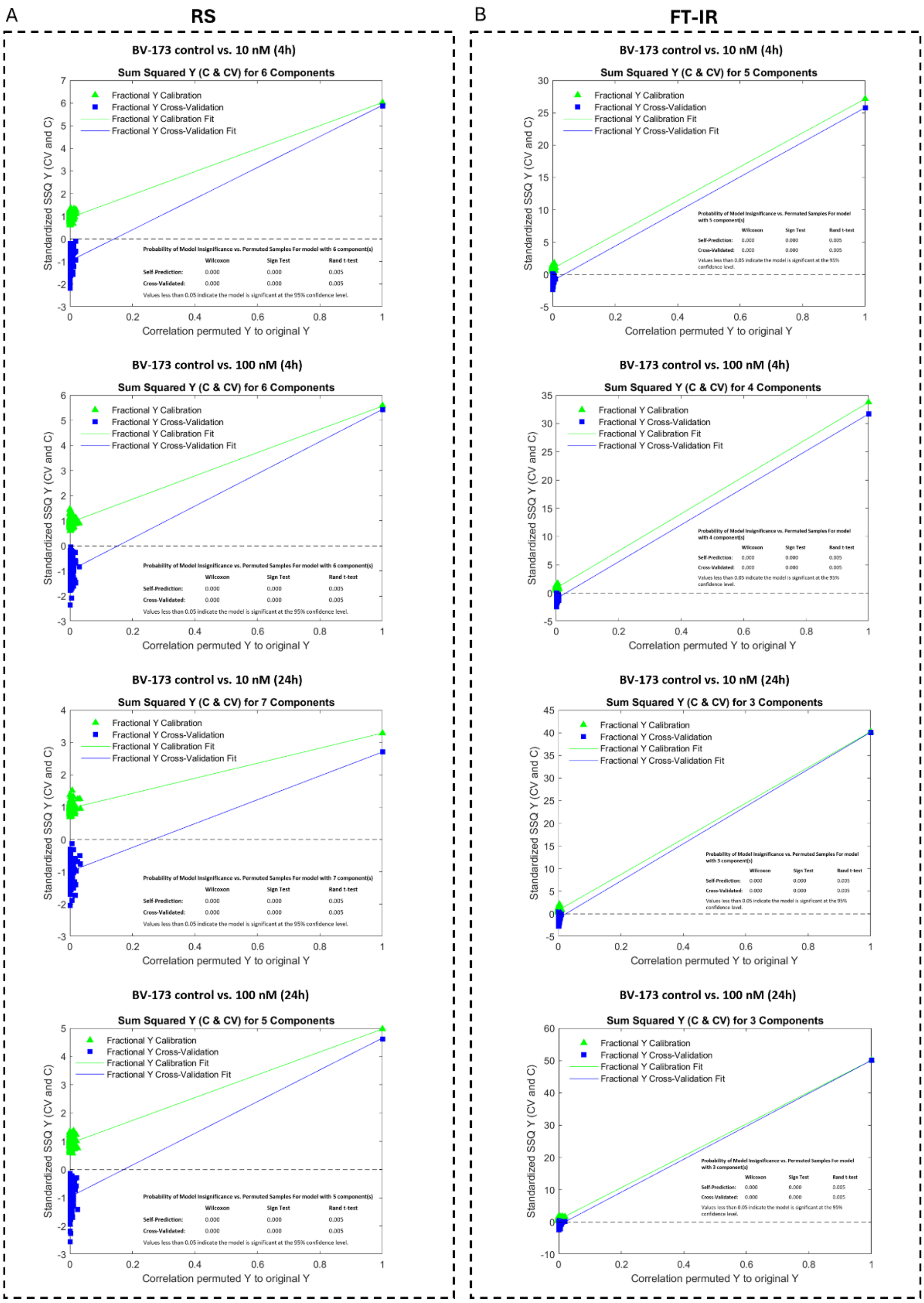

Fig. S4 Permutation test for PLS-DA of RS (A) and FT-IR (B) spectra for an in vitro model of BCR-ABL1 cells based on the BV-173 cell line. Plot of standardized Sum Square Y vs. correlation for 200 permutations. The tables show the probability of the model being insignificant. Values less than 0.05 indicate the model is significant at the 95% confidence level. The presented values indicate that the observed separation exceeds chance-level expectations.

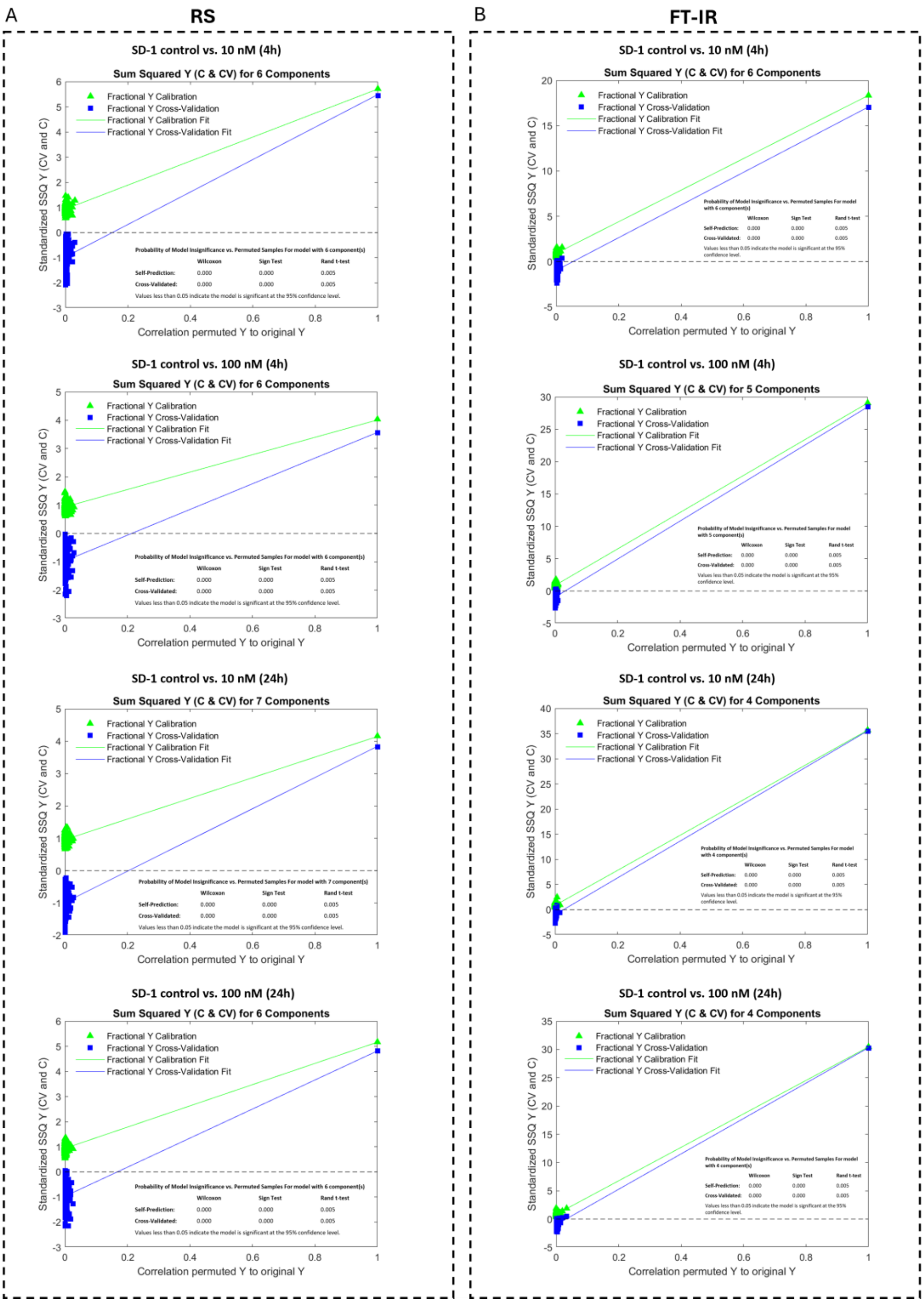

Fig. S5 Permutation test for PLS-DA of RS (A) and FT-IR (B) spectra for an in vitro model of BCR-ABL1 cells based on the SD-1 cell line. Plot of standardized Sum Square Y vs. correlation for 200 permutations. The tables show the probability of the model being insignificant. Values less than 0.05 indicate the model is significant at the 95% confidence level. The presented values indicate that the observed separation exceeds chance-level expectations.

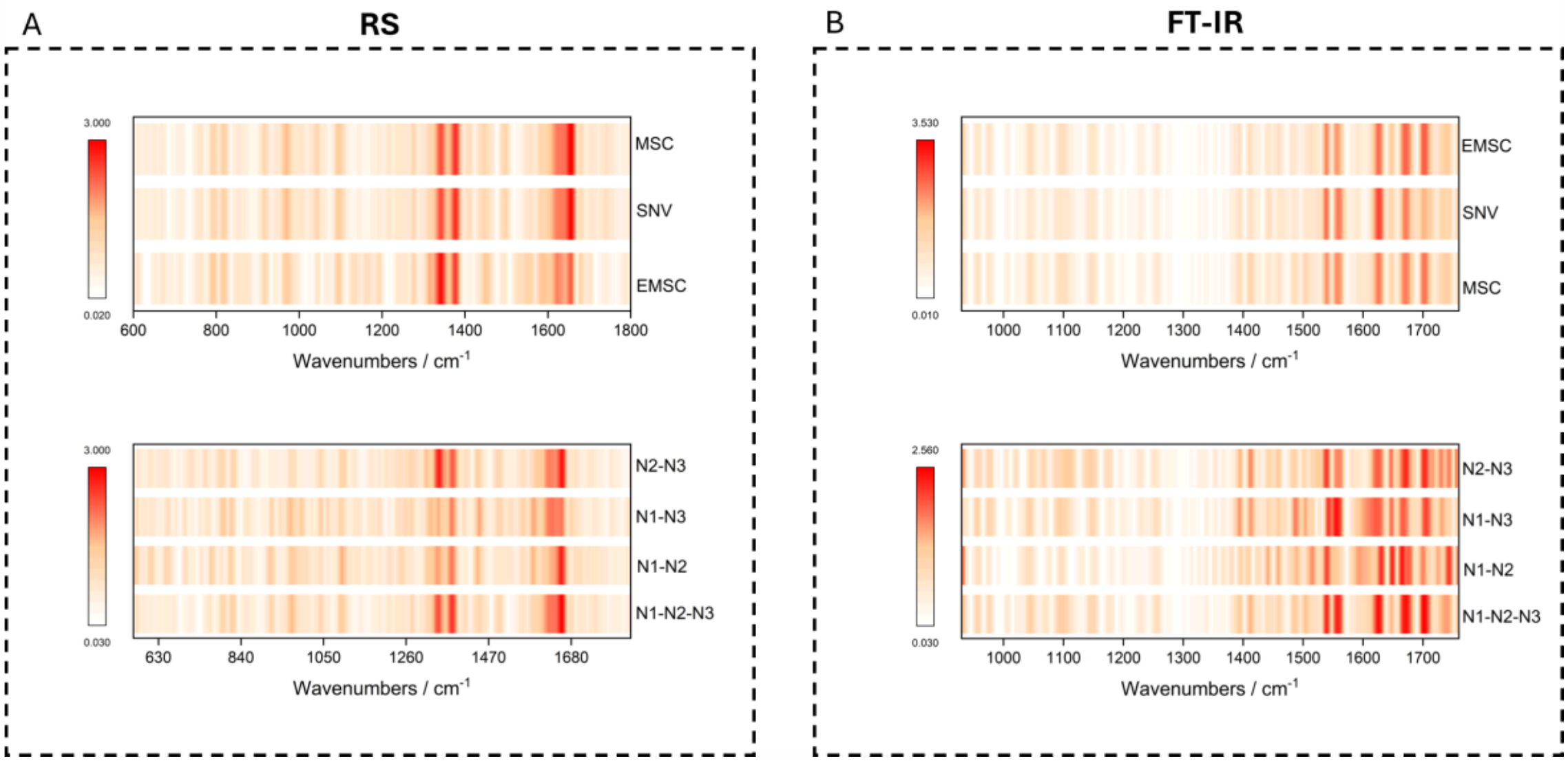

Fig. S6 Heat maps of VIP values for PLS-DA models of RS (A) and FT-IR (B) spectra of the SD-1 and BV-173 cells in different normalization variants (EMSC, MSC, SNV) and data sets.

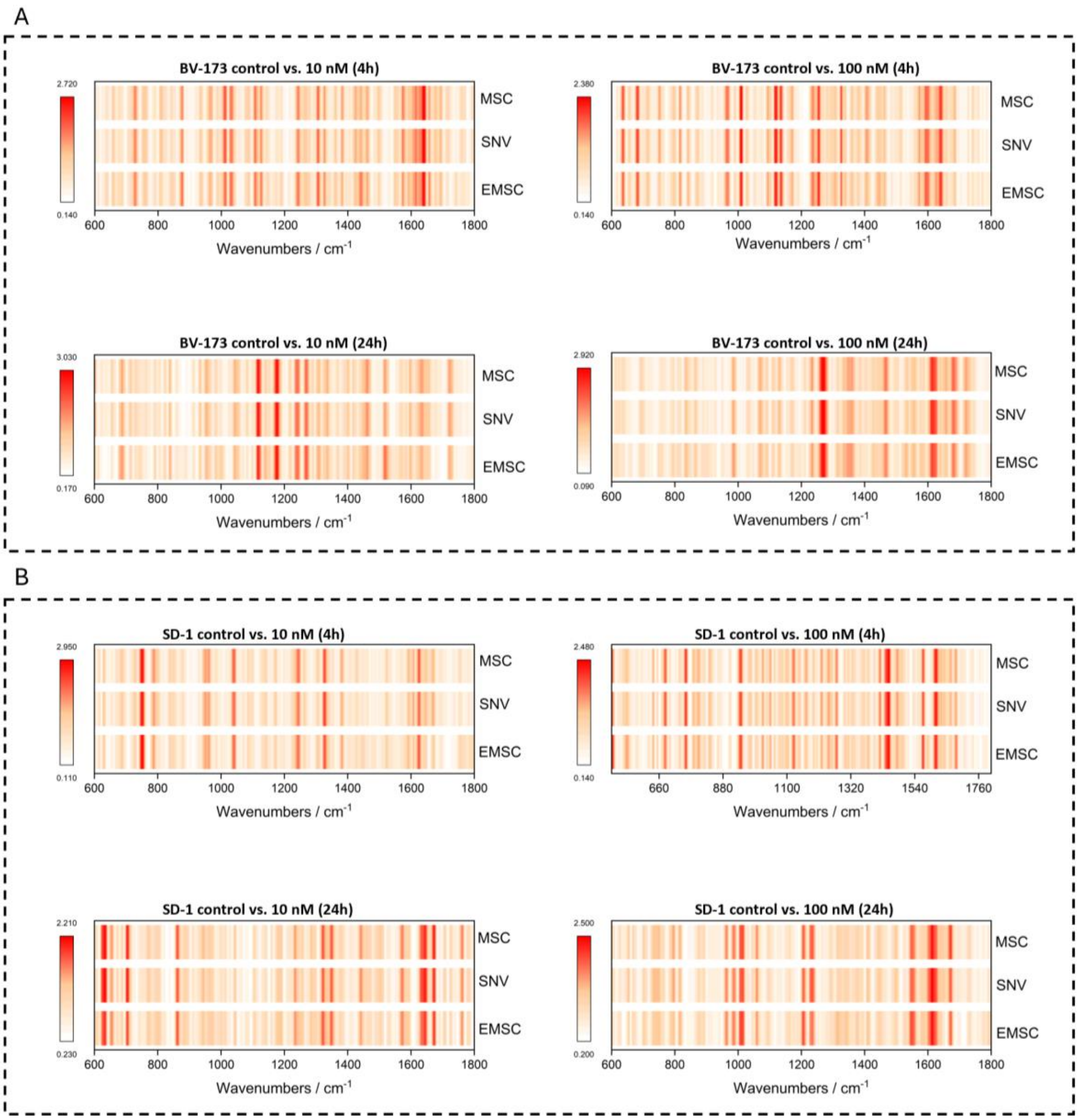

Fig. S7 Heat maps of VIP values for PLS-DA of RS spectra for an in vitro model of BCR-ABL1 cells based on the BV-173 (A) and SD-1 (B) cell line in different normalization variants (EMSC, MSC, SNV).

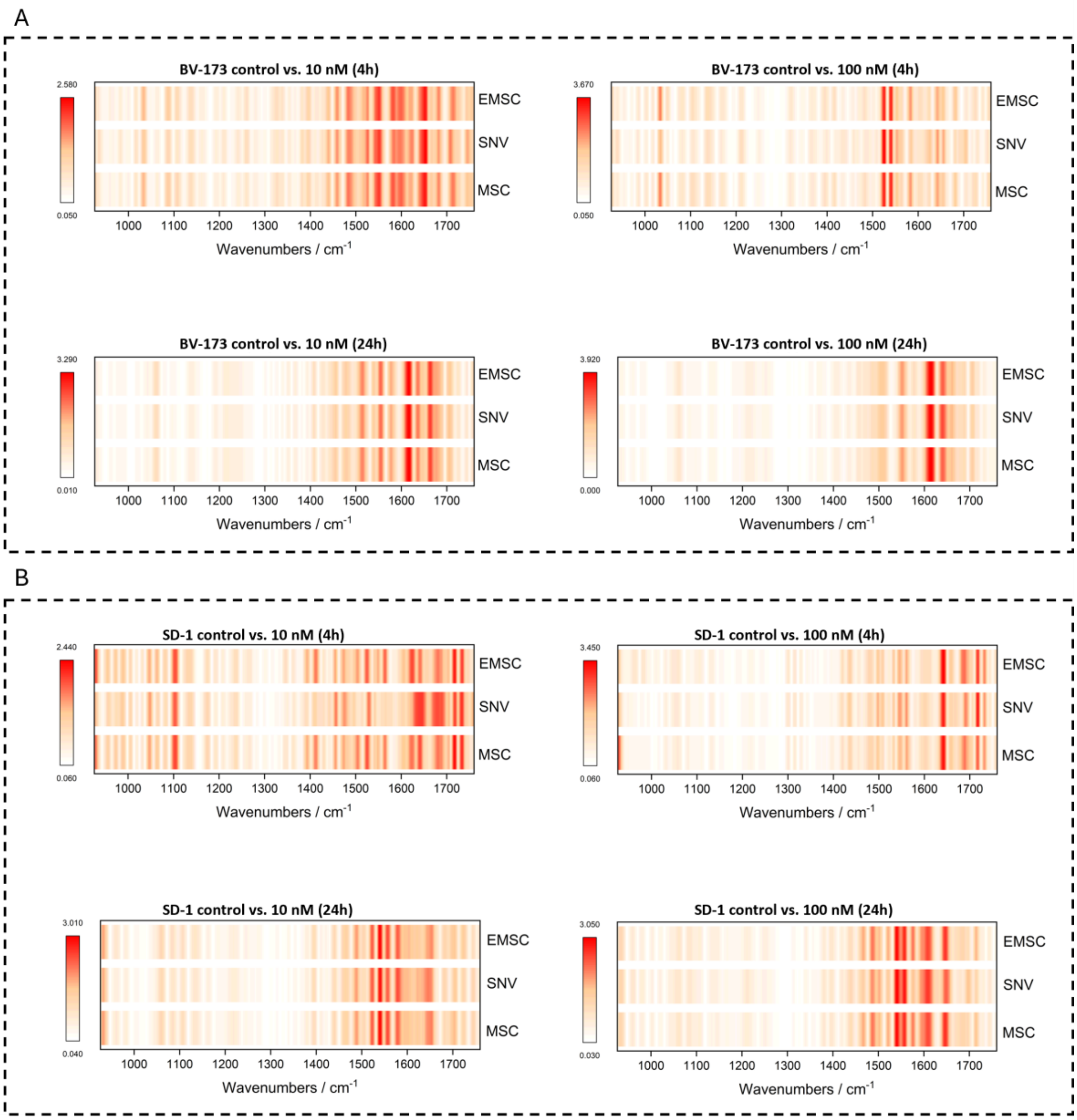

Fig. S8 Heat maps of VIP values for PLS-DA of FT-IR spectra for an in vitro model of BCR-ABL1 cells based on the BV-173 (A) and SD-1 (B) cell line in different normalization variants (EMSC, MSC, SNV).

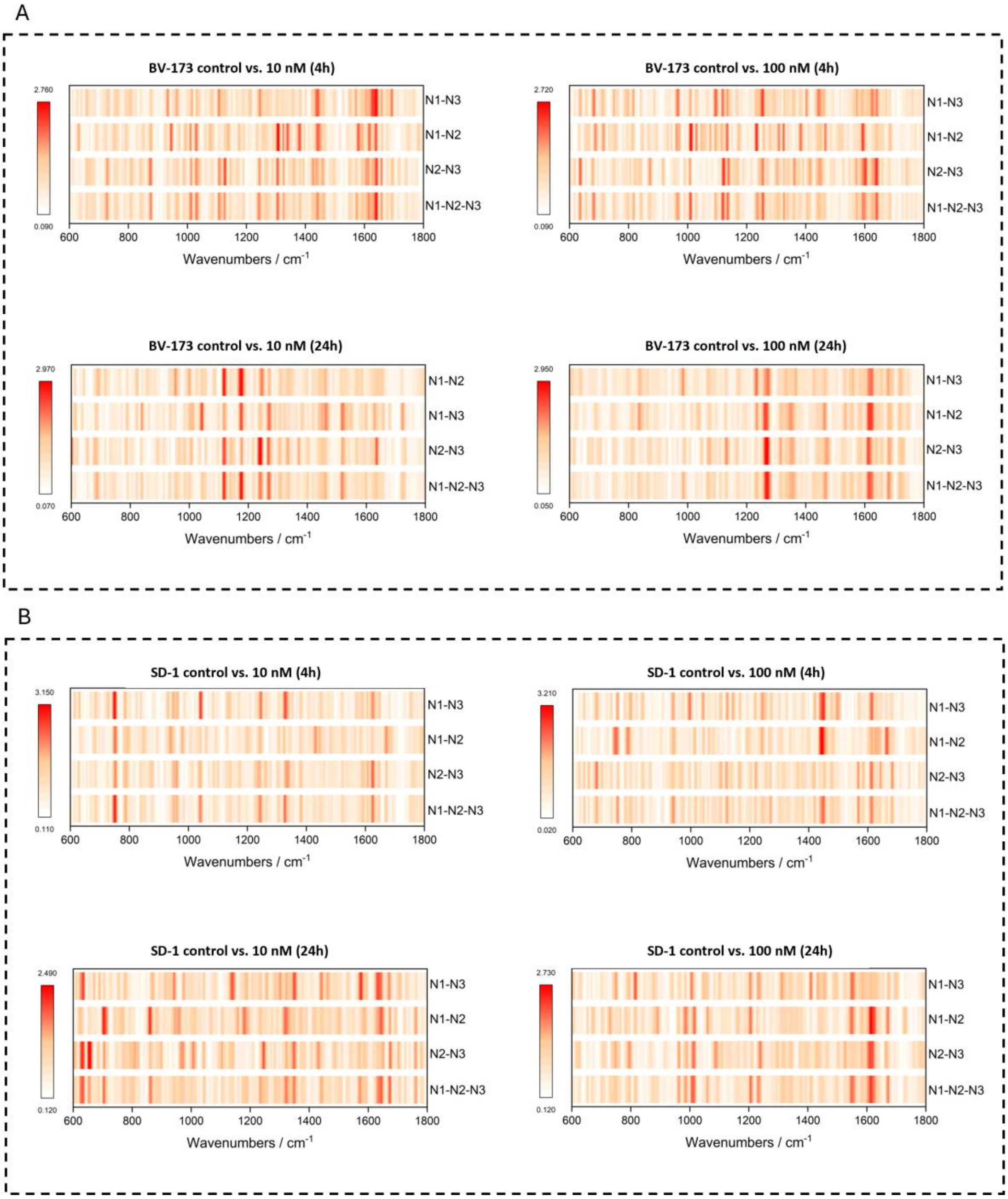

Fig. S9 Heat maps of VIP values for PLS-DA of RS spectra for an in vitro model of BCR-ABL1 cells based on the BV-173 (A) and SD-1 (B) cell line in different data sets.

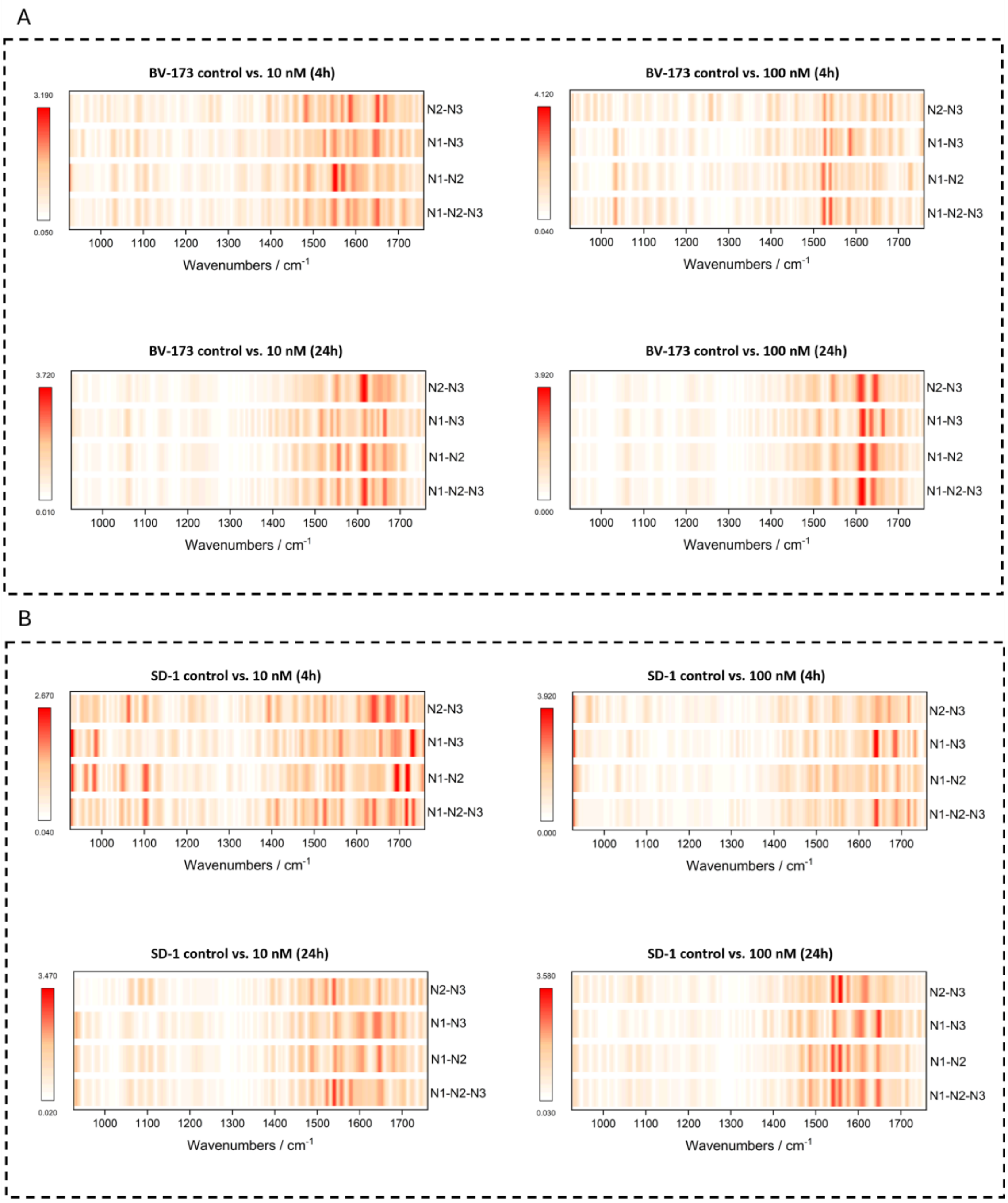

Fig. S10 Heat maps of VIP values for PLS-DA of FT-IR spectra for an in vitro model of BCR-ABL1 cells based on the BV-173 (A) and SD-1 (B) cell line in different data sets.

+

3. Band assignment table

Tab. S2 Assignments of the Raman bands observed in the spectra of studied samples[4–20]

Band / cm^{-1}	Assignment
1760-1720	C=O symmetric stretching in lipids and esters
1695	nucleic acids
1680-1635	Amide I (proteins)
1660	Amide I, C=C stretching (proteins)
1625/1620	Trp, Tyr
1610	C=C in-plane bending mode of Phe, Tyr
1600	C=C in-plane bending mode of Phe
1595-1575	DNA bases
1570	Trp
1556	Phe, Tyr
1490-1470	ring breathing of G, A; C-H deformation mode in proteins
1460-1450	CH_2 deformation in proteins
1440-1450	CH_2 deformation in proteins
1420-1400	C-H deformation in proteins
1380/1375	DNA bases
1355-1360	Trp (CH_3 wagging/indole deformation), DNA bases
1340/1350	DNA bases; Ser, Thr δ(C–H)
1315/1306	twisting mode of (CH_2) in lipids; porphyrin ring breathing mode in haemoproteins
1297	fatty acids and triacylglycerols; twisting mode of (CH_2/CH_3)
1289	nucleic acids
1255-1270	lipids: deformation (=CH)
1275-1220	amide III (C-N stretching, N-H bending)
1205-1210	C-C_6H_5 stretching mode of Tyr and Phe
1175-1185	Tyr, Phe, protein C-H bending
1170	C-C stretching in fatty acids
1137-1103	protein: C–N, C–C stretching
1130-1135	ν(C–C); porphyrin ring breathing mode in haemoproteins
1095	PO_2^- symmetric stretching
1080	C-C stretching in fatty acids; PO_2^- stretching of the DNA backbone
1070	lipids (gauche in acyl backbone); stretching mode of (C-C)
1060	lipids: chain C–C
1045-1030	C-N stretching, Phe
1016/1010	ring breathing of Phe
988	protein C-C stretching
968	ribose vibration (RNA)
957	protein skeletal vibration
936	protein C-C stretching
915	ribose vibration (RNA)
888	CH_2 rocking deformation mode
876	Trp
866	unsaturated fatty acids and triacylglycerols; stretching mode of (C-O-O)
860	Tyr
855	ring breathing in Tyr (Fermi doublet)
843	C-C stretching mode of glucose
837/825	PO_2^- asymmetric stretching, Tyr (Fermi doublet)
816/809	RNA
800-770	ring breathing of C, U, T
755-765	Trp
750	porphyrin ring breathing mode in haemoproteins
740	T
728	ring breathing of A
713	(N+(CH_3)$_3$) stretching of the choline group
701	cholesterol
690-670	G, T, proteins: C-C twisting
657	ring breathing of Phe and Tyr
650/640	proteins: C-S stretching, Tyr: C-C twisting

630	proteins: C-C twisting, Phe

A – adenine; C – cytosine; G – guanine; U – uracil; T – thymine; Tyr – tyrosine; Phe – phenylalanine; Trp - tryptophan

Tab. S3 Assignments of the FT-IR bands observed in the spectra of the studied samples[5,21–34]

Band / cm-1	Assignment
1735-1730	lipids, ester: stretching mode of C=O
1720-1715	fatty acids: stretching mode of C=O
1705	nitrogenous bases of nucleic acids
1690-1673	β-turns in proteins (amide I)
1667-1642	α-helices in proteins (amide I)
1630-1615	β-sheets in proteins: intracellular aggregations of cellular proteins (amide I)
1600	aromatic ring C=C stretching: DNA bases, Phe, Tyr
1595-1578	aromatic ring C-C stretching in proteins
1565-1530	N-H deformation vibration and C-N stretching (amide II)
1525	Tyr (proteins)
1503	CH in-plane bending
1495-1482	aromatic ring C-C stretch in proteins
1467-1445	CH_3, CH_2 bending modes in proteins
1438-1445	CH_2 bending in lipids
1412	stretching C–N, deformation N–H, deformation C–H (proteins)
1392	symmetric CH_3 bending of the methyl groups in proteins
1325-1315	amide III (protein)
1260-1240	PO_2^- asymmetric stretching of nucleic acids
1175-1170	C-O stretching of Ser, Thr, Tyr
1150-1130	C-O stretching mode of the carbohydrates
1095-1070	PO_2^- symmetric stretching of nucleic acids, phospholipids, proteins
1060-1022	C-O stretching: polysaccharides, DNA
1007	stretching of C-O deoxyribose
990-986	phosphate vibrations in nucleic acids, carbohydrate ring modes in RNA
978	RNA ribose-phosphate skeletal motions
960	C-O deoxyribose DNA, symmetric phosphate vibration (PO_4^{3-}), DNA backbone
933	Z-DNA

4. Independent replicate-level validation

To evaluate robustness of treatment-associated spectral trends and address potential information leakage associated with single-cell cross-validation, additional leave-one-replicate-out validation was performed. Models were constructed using two biological replicates and tested on the third independent replicate. Regression vectors and VIP heatmaps were compared between validation strategies to assess consistency of discriminative spectral regions.

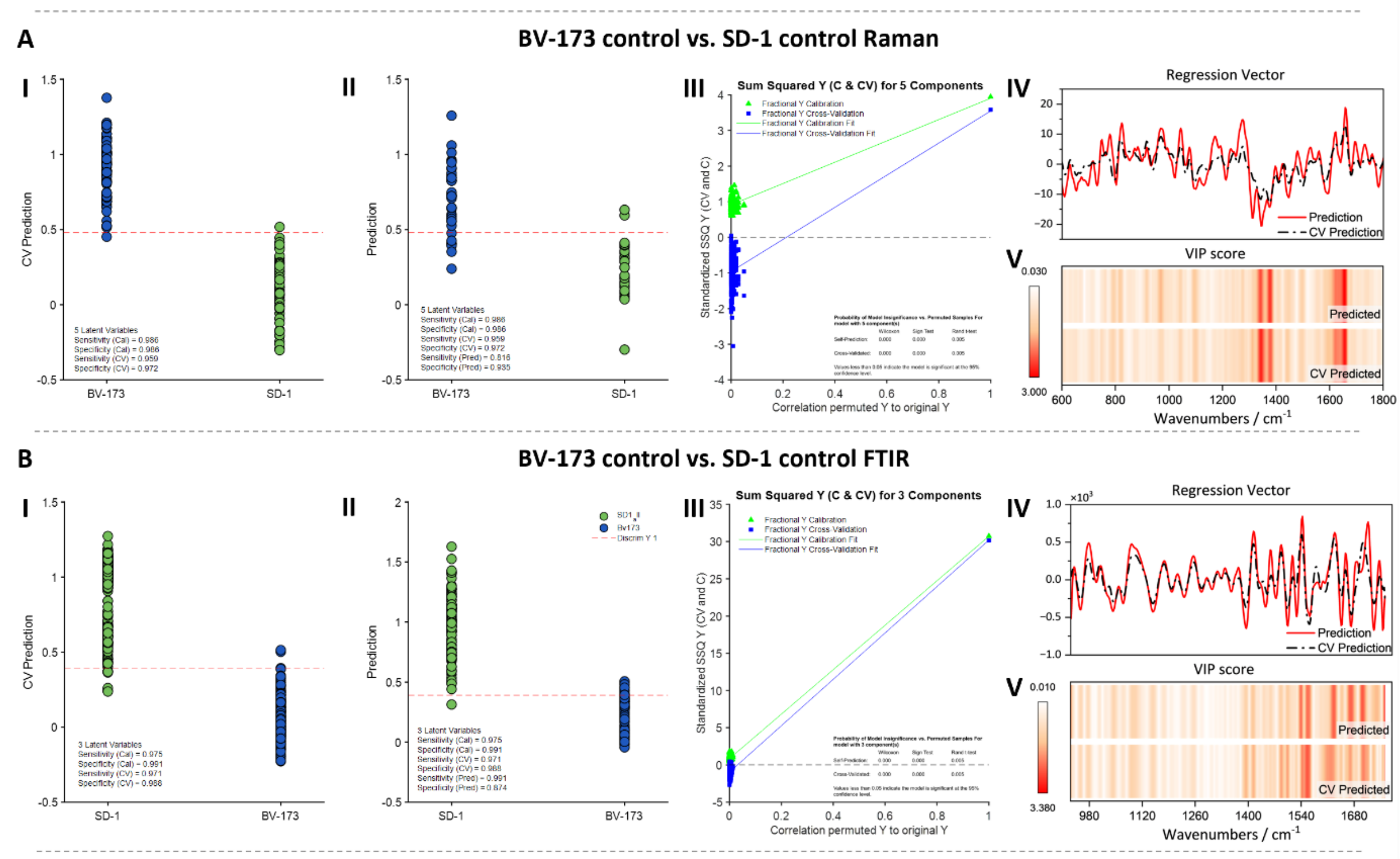

Fig. S11 PLS-DA results for RS (A) and FT-IR (B) spectra of control SD-1 and BV-173 cells. (I) PLS-DA results obtained using Venetian blinds cross-validation (*CV Prediction*) and (II) leave-one-replicate-out prediction (*Prediction*). For the leave-one-replicate-out approach, the PLS-DA model was constructed using two independent biological replicates, while the third biological replicate was used exclusively for external prediction. (III) Permutation test for PLS-DA showing the plot of standardized Sum Square Y versus correlation for 200 permutations. The presented results indicate that the observed class separation exceeded chance-level expectations. (IV) Comparison of regression vectors obtained for the leave-one-replicate-out prediction model (*Prediction*) and the PLS-DA model constructed using all three biological replicates presented in the main manuscript (*CV Prediction*). (V) Heatmaps of VIP values obtained for the leave-one-replicate-out prediction model (*Prediction*) and the PLS-DA model constructed using all three biological replicates presented in the main manuscript (*CV Prediction*).

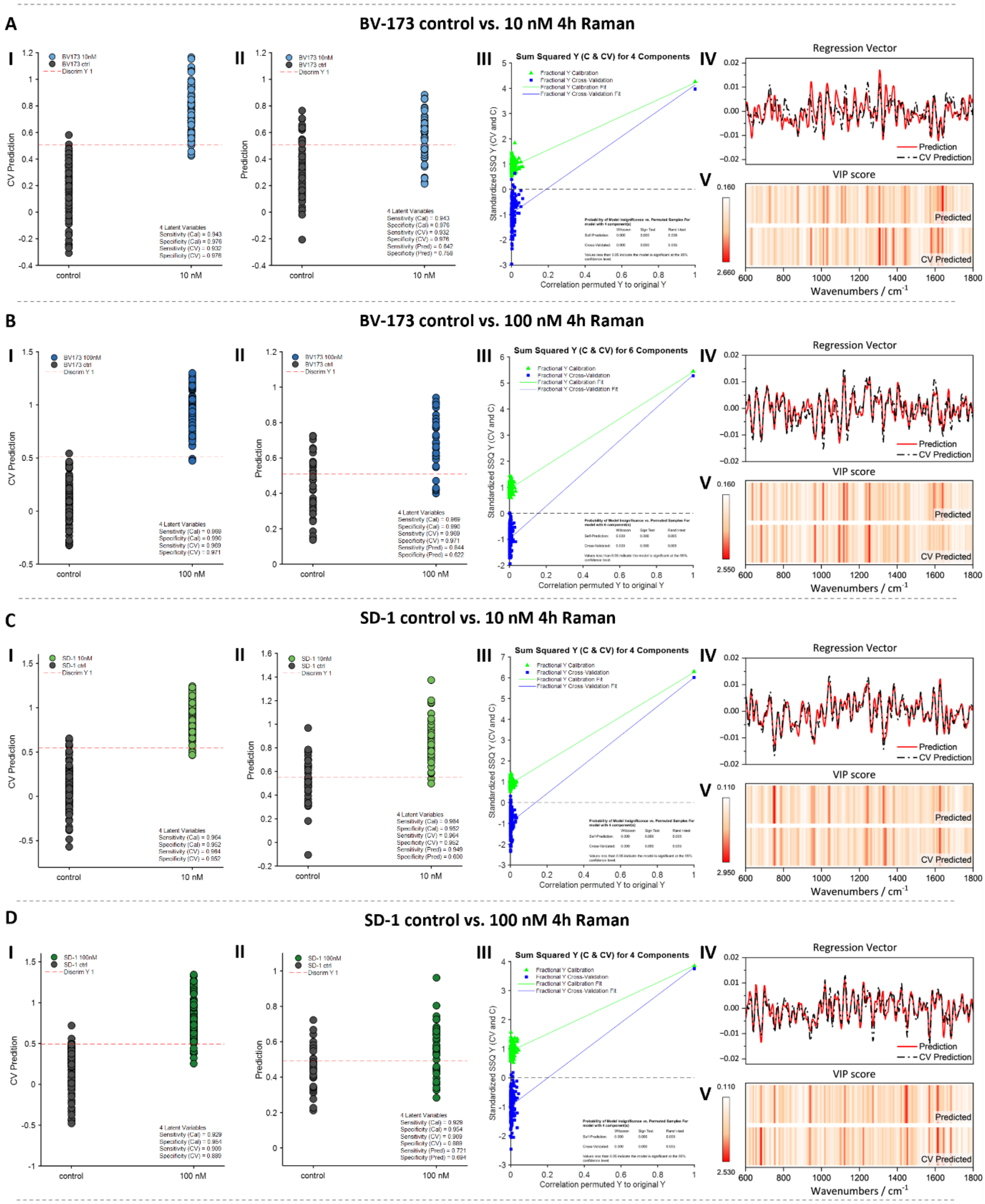

Fig. S12 PLS-DA results for RS spectra of the BV-173 cell line (A – control vs. 10 nM, B – control vs. 100 nM) and the SD-1 cell line (C – control vs. 10 nM, D – control vs. 100 nM) after 4 h incubation with VEN. (I) PLS-DA results obtained using Venetian blinds cross-validation (*CV Prediction*) and (II) leave-one-replicate-out prediction (*Prediction*). For the leave-one-replicate-out approach, the PLS-DA model was constructed using two independent biological replicates, with the third replicate used exclusively for external prediction. (III) Permutation test for PLS-DA showing the plot of standardized Sum Square Y versus correlation for 200 permutations. The presented results indicate that the observed class separation exceeded chance-level expectations. (IV) Comparison of regression vectors obtained for the leave-one-replicate-out prediction model (*Prediction*) and the PLS-DA model constructed using all three biological replicates presented in the main manuscript (*CV Prediction*). (V) Heatmaps of VIP values obtained for the leave-one-replicate-out prediction model (*Prediction*) and the PLS-DA model constructed using all three biological replicates presented in the main manuscript (*CV Prediction*).

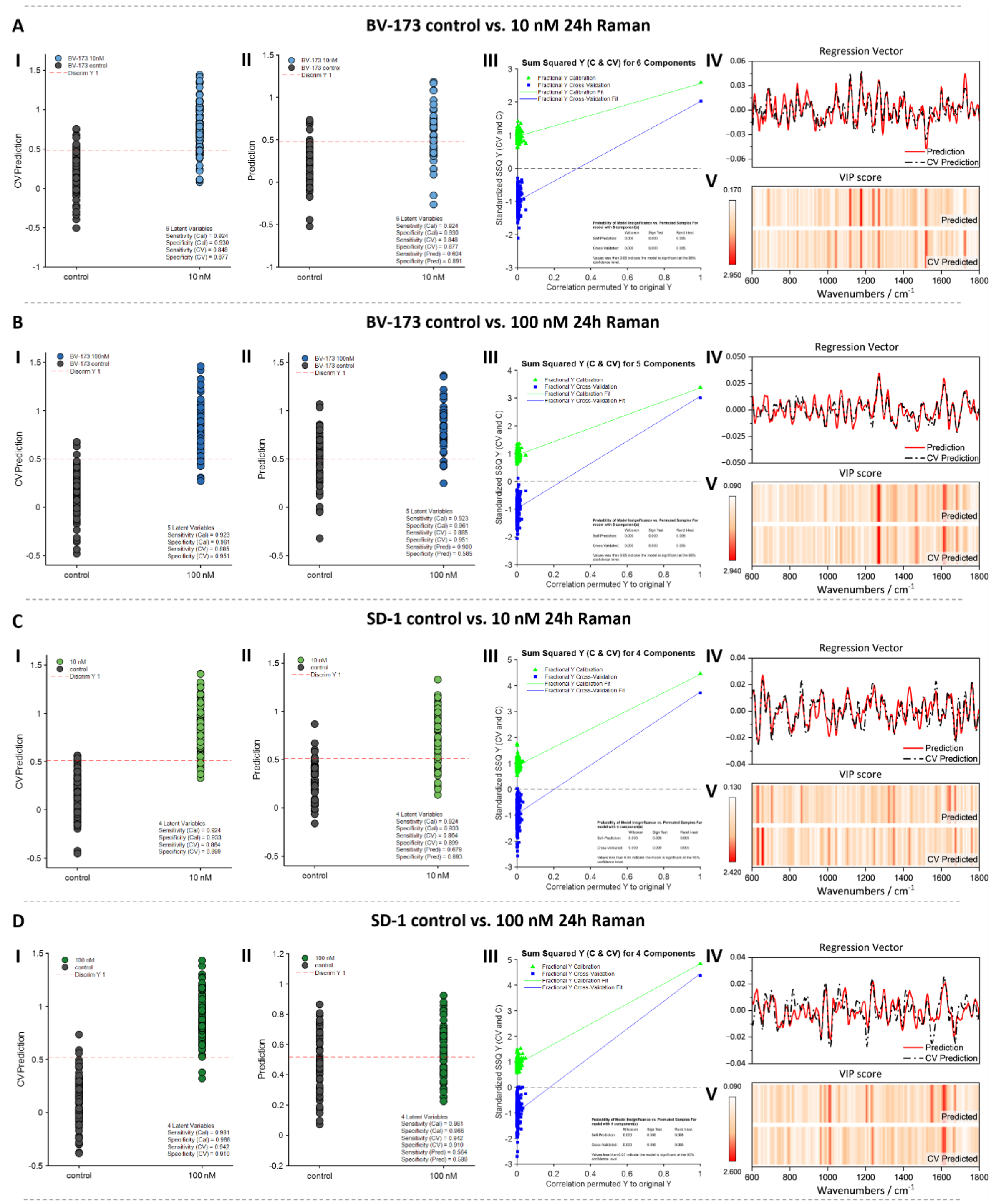

Fig. S13 PLS-DA results for RS spectra of the BV-173 cell line (A – control vs. 10 nM, B – control vs. 100 nM) and the SD-1 cell line (C – control vs. 10 nM, D – control vs. 100 nM) after 24 h incubation with VEN. (I) PLS-DA results obtained using Venetian blinds cross-validation (*CV Prediction*) and (II) leave-one-replicate-out prediction (*Prediction*). For the leave-one-replicate-out approach, the PLS-DA model was constructed using two independent biological replicates, while the third biological replicate was used exclusively for external prediction. (III) Permutation test for PLS-DA showing the plot of standardized Sum Square Y versus correlation for 200 permutations. The presented results indicate that the observed class separation exceeded chance-level expectations. (IV) Comparison of regression vectors obtained for the leave-one-replicate-out prediction model (*Prediction*) and the PLS-DA model constructed using all three biological replicates presented in the main manuscript (*CV Prediction*). (V) Heatmaps of VIP values obtained for the leave-one-replicate-out prediction model (*Prediction*) and the PLS-DA model constructed using all three biological replicates presented in the main manuscript (*CV Prediction*).

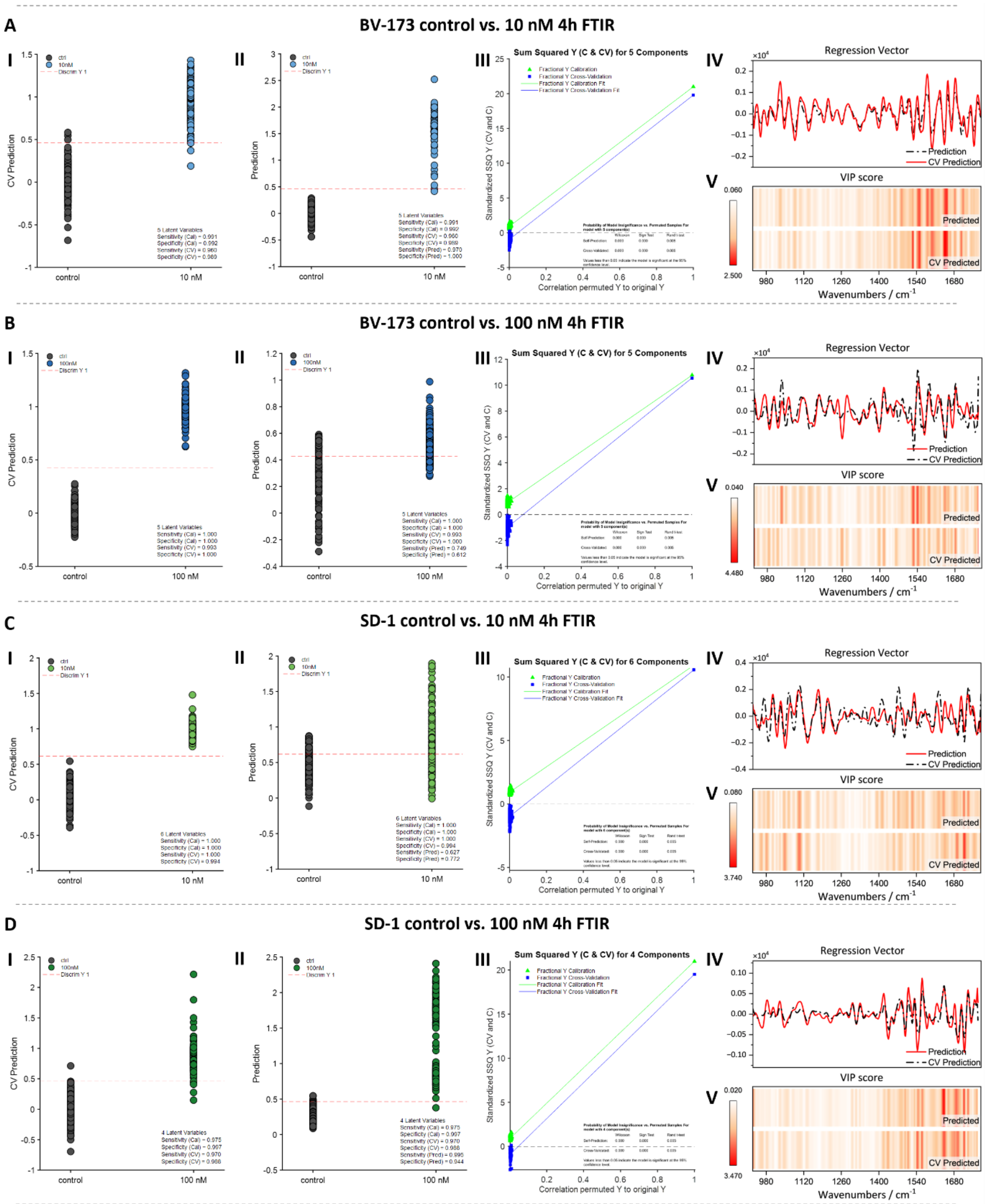

Fig. S14 PLS-DA results for FT-IR spectra of the BV-173 cell line (A – control vs. 10 nM, B – control vs. 100 nM) and the SD-1 cell line (C – control vs. 10 nM, D – control vs. 100 nM) after 4 h incubation with VEN. (I) PLS-DA results obtained using Venetian blinds cross-validation (*CV Prediction*) and (II) leave-one-replicate-out prediction (*Prediction*). For the leave-one-replicate-out approach, the PLS-DA model was constructed using two independent biological replicates, with the third replicate used exclusively for external prediction. (III) Permutation test for PLS-DA showing the plot of standardized Sum Square Y versus correlation for 200 permutations. The presented results indicate that the observed class separation exceeded chance-level expectations. (IV) Comparison of regression vectors obtained for the leave-one-replicate-out prediction model (*Prediction*) and the PLS-DA model constructed using all three biological replicates presented in the main manuscript (*CV Prediction*). (V) Heatmaps of VIP values obtained for the leave-one-replicate-out prediction model (*Prediction*) and the PLS-DA model constructed using all three biological replicates presented in the main manuscript (*CV Prediction*).

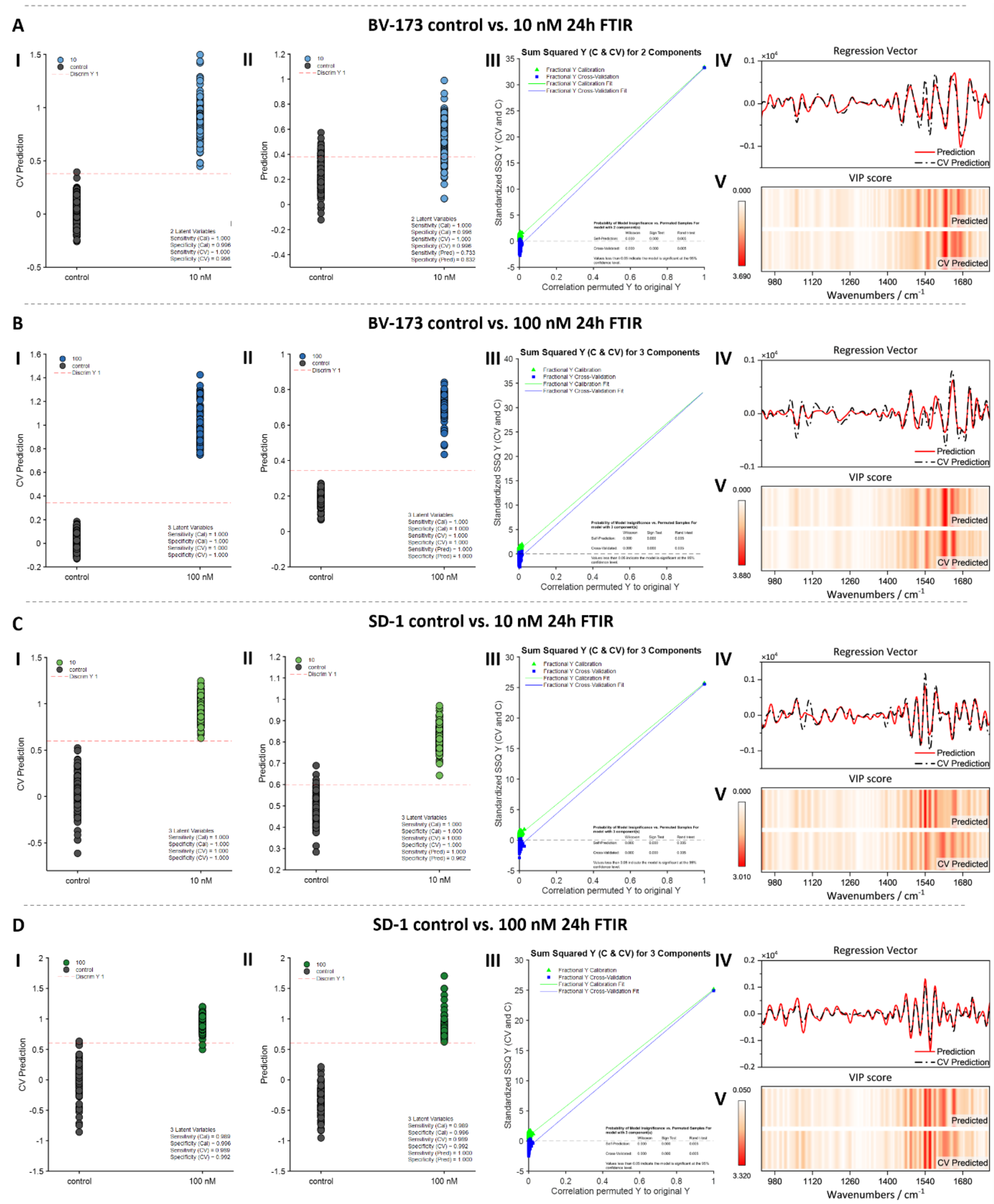

Fig. S15 PLS-DA results for FT-IR spectra of the BV-173 cell line (A – control vs. 10 nM, B – control vs. 100 nM) and the SD-1 cell line (C – control vs. 10 nM, D – control vs. 100 nM) after 24 h incubation with VEN. (I) PLS-DA results obtained using Venetian blinds cross-validation (*CV Prediction*) and (II) leave-one-replicate-out prediction (*Prediction*). For the leave-one-replicate-out approach, the PLS-DA model was constructed using two independent biological replicates, while the third biological replicate was used exclusively for external prediction. (III) Permutation test for PLS-DA showing the plot of standardized Sum Square Y versus correlation for 200 permutations. The presented results indicate that the observed class separation exceeded chance-level expectations. (IV) Comparison of regression vectors obtained for the leave-one-replicate-out prediction model (*Prediction*) and the PLS-DA model constructed using all three biological replicates presented in the main manuscript (*CV Prediction*). (V) Heatmaps of VIP values obtained for the leave-one-replicate-out prediction model (*Prediction*) and the PLS-DA model constructed using all three biological replicates presented in the main manuscript (*CV Prediction*).

5. References

1. Kristian, N. & Kohler, A. Chemometrics and Intelligent Laboratory Systems Extended multiplicative signal correction in vibrational spectroscopy , a tutorial. *Chemom. Intell. Lab. Syst.* **117**, 92–99 (2012).

2. Warringer, J. *et al.* Reducing Inter-replicate Variation in Fourier Transform Infrared Spectroscopy by Extended Multiplicative Signal Correction. **63**, 296–305 (2009).

3. Féré, M. *et al.* Vibrational Spectroscopy Focus on pre-processing step to ensure the clinical transferability of Raman data acquired on lymphocytes in di ff erent experimental and instrumental conditions. *Vib. Spectrosc.* **103**, 102931 (2019).

4. Adamczyk, A. *et al.* Raman classification of selected subtypes of acute lymphoblastic leukemia (ALL). *Analyst* **149**, 571–581 (2023).

5. Augustyniak, K. *et al.* Molecular tracking of interactions between progenitor and endothelial cells via Raman and FTIR spectroscopy imaging: a proof of concept of a new analytical strategy for in vitro research (Cellular and Molecular Life Sciences, 80, 11, (2023).

6. Bankapur, A., Zachariah, E., Chidangil, S., Valiathan, M. & Mathur, D. Raman tweezers spectroscopy of live, single red and white blood cells. *PLoS One* **5**, (2010).

7. Chan, J. W. *et al.* Micro-raman spectroscopy detects individual neoplastic and normal hematopoietic cells. *Biophys. J.* **90**, 648–656 (2006).

8. Czamara, K. *et al.* Raman spectroscopy of lipids: A review. *J. Raman Spectrosc.* **46**, 4-20 (2015).

9. De Gelder, J., De Gussem, K., Vandenabeele, P. & Moens, L. Reference database of Raman spectra of biological molecules. *J. Raman Spectrosc.* **38**, 1133–1147 (2007).

10. Krafft, C., Knetschke, T., Siegner, A., Funk, R. H. W. & Salzer, R. Mapping of single cells by near infrared Raman microspectroscopy. *Vib. Spectrosc.* **32**, 75–83 (2003).

11. Leszczenko, P. *et al.* Towards raman-based screening of acute lymphoid leukemia-type b (B-all) subtypes. *Cancers (Basel).* **13**, 1–21 (2021).

12. Leszczenko, P. *et al.* Advancing triage of acute lymphoblastic leukaemia subtypes diagnosis: label-free Raman spectroscopy for precise single-cell phenotyping and subtype classification. *Analyst* (2024) doi:10.1039/D4AN00956H.

13. Lyng, F. M., Ramos, I. R. M., Ibrahim, O. & Byrne, H. J. Vibrational microspectroscopy for cancer screening. *Appl. Sci.* **5**, 23–35 (2015).

14. Managò, S. *et al.* A reliable Raman-spectroscopy-based approach for diagnosis, classification and follow-up of B-cell acute lymphoblastic leukemia. *Sci. Rep.* **6**, 1–13 (2016).

15. Managò, S., Zito, G. & De Luca, A. C. Raman microscopy based sensing of leukemia cells: A review. *Opt. Laser Technol.* **108**, 7–16 (2018).

16. Movasaghi, Z., Rehman, S. & Rehman, I. U. Raman spectroscopy of biological tissues. *Appl. Spectrosc. Rev.* **42**, 493–541 (2007).

17. Nawaz, H., Garcia, A., Meade, A. D., Lyng, F. M. & Byrne, H. J. Raman micro spectroscopy study of the interaction of vincristine with A549 cells supported by expression analysis of bcl-2 protein. *Analyst* **138**, 6177–6184 (2013).

18. Notingher, I. *et al.* In situ Characterisation of Living Cells by Raman Spectroscopy. *Spectroscopy* **16**, 43–51 (2002).

19. Notingher, I. *et al.* Discrimination between ricin and sulphur mustard toxicity in vitro using Raman spectroscopy. *J. R. Soc. Interface* **1**, 79–90 (2004).

20. Rygula, A. *et al.* Raman spectroscopy of proteins: A review. *J. Raman Spectrosc.* **44**, 1061-1076(2013).

21. Baran, Y., Ceylan, C. & Camgoz, A. The roles of macromolecules in imatinib resistance of chronic myeloid leukemia cells by Fourier transform infrared spectroscopy. *Biomed. Pharmacother.* **67**, 221–227 (2013).

22. Severcan, F., Bozkurt, O., Gurbanov, R. & Gorgulu, G. FT-IR spectroscopy in diagnosis of diabetes in rat animal model. *J. Biophotonics* **3**, 621–631 (2010).

23. Sheng, D. *et al.* Distinction of leukemia patients' and healthy persons' serum using FTIR spectroscopy. *Spectrochim. Acta - Part A Mol. Biomol. Spectrosc.* **101**, 228–232 (2013).

24. Travo, A. *et al.* Basis of a FTIR spectroscopy methodology for automated evaluation of Akt kinase inhibitor on leukemic cell lines used as model. *Anal. Bioanal. Chem.* **404**, 1733–1743 (2012).

25. Zelig, U. *et al.* Pre-screening and follow-up of childhood acute leukemia using biochemical infrared analysis of peripheral blood mononuclear cells. *Biochim. Biophys. Acta - Gen. Subj.* **1810**, 827–835 (2011).

26. Zucchiatti, P. *et al.* Contribution of Ribonucleic Acid (RNA) to the fourier transform infrared (FTIR) Spectrum of eukaryotic cells. *Anal. Chem.* **88**, 12090–12098 (2016).

27. Benedetti, E., Bramanti, E., Papineschi, F., Rossi, I. & Benedetti, E. Determination of the relative amount of nucleic acids and proteins in leukemic and normal lymphocytes by means of fourier transform infrared microspectroscopy. *Appl. Spectrosc.* **51**, 792–797 (1997).

28. Dovbeshko, G. I. *et al.* Surface enhanced IR absorption of nucleic acids from tumor cells: FTIR reflectance study. *Biopolym. - Biospectroscopy Sect.* **67**, 470–486 (2002).

29. Dovbeshko, G. I., Gridina, N. Y., Kruglova, E. B. & Pashchuk, O. P. FTIR spectroscopy studies of nucleic acid damage. *Talanta* **53**, 233–246 (2000).

30. GASPARRI, F. & MUZIO, M. Monitoring of apoptosis of HL60 cells by Fourier-transform infrared spectroscopy. *Biochem. J.* **369**, 239–248 (2003).

31. Gault, N. & Lefaix, J. L. Infrared microspectroscopic characteristics of radiation-induced apoptosis in human lymphocytes. *Radiat. Res.* **160**, 238–250 (2003).

32. Li, Y. *et al.* Remodeling Chromatin Induces Z-DNA Conformation Detected through Fourier Transform Infrared Spectroscopy. *Anal. Chem.* **92**, 14452–14458 (2020).

33. Movasaghi, Z., Rehman, S. & Rehman, I. U. Fourier transform infrared (FTIR) spectroscopy of biological tissues. *Appl. Spectrosc. Rev.* **43**, 134–179 (2008).

34. Sahu, R. K., Mordechai, S. & Manor, E. Nucleic acids absorbance in Mid IR and its effect on diagnostic variates during cell division: A case study with lymphoblastic cells. *Biopolymers* **89**, 993–1001 (2008).
